# Secondary Metabolites from the Root Rot Biocontrol Fungus *Phlebiopsis gigantea*

**DOI:** 10.3390/molecules23061417

**Published:** 2018-06-12

**Authors:** David Kälvö, Audrius Menkis, Anders Broberg

**Affiliations:** 1 Department of Molecular Sciences, Uppsala BioCenter, Swedish University of Agricultural Sciences, P.O. Box 7015, SE-75007 Uppsala, Sweden; david.kalvo@outlook.com; 2 Department of Forest Mycology and Plant Pathology, Uppsala BioCenter, Swedish University of Agricultural Sciences, P.O. Box 7026, SE-75007 Uppsala, Sweden; Audrius.Menkis@slu.se

**Keywords:** *Phlebiopsis gigantea*, phanerochaetaceae, biocontrol, *Heterobasidion* spp., secondary metabolite characterization, cyclopentanoids, *p*-terphenyls

## Abstract

Three cyclopentanoids (phlebiopsin A–C), one glycosylated *p*-terphenyl (methyl-terfestatin A), and *o*-orsellinaldehyde were isolated from the biocontrol fungus *Phlebiopsis gigantea*, and their structures were elucidated by 1D and 2D NMR spectroscopic analysis, as well as by LC-HRMS. The biological activity of the compounds against the root rot fungus *Heterobasidion occidentale*, as well as against *Fusarium oxysporum* and *Penicillium canescens*, was also investigated, but only *o*-orsellinaldehyde was found to have any antifungal activity in the concentration range tested.

## 1. Introduction

Root and stem rot diseases of conifers cause huge economic losses, and are substantial problems for forestry. Several fungal species can cause root and stem rot, but several basidiomycete species of the genus *Heterobasidion* are, by far, the most widespread and destructive pathogens [1]. Their initial route of spread and infection is by airborne basidiospores that infect freshly cut stump surfaces or wounds on the roots or stem. Following stump colonization, the pathogen grows down through the root system, and can spread efficiently to adjacent healthy trees by root-to-root contact [2].

*Phlebiopsis gigantea* (Fr.) Jülich, is a saprophytic basidiomycete fungus used as a biocontrol agent to prevent *Heterobasidion* root rot. Biocontrol with *P. gigantea* is accomplished by applying arthroconidia (oidia) of the fungus to the stump surface soon after felling of a tree. Thor and Stenlid [3] have shown that this biocontrol treatment is highly efficient, and may reduce *Heterobasidion* stump infections by 88–99% compared with untreated stumps. Rotstop^®^, a commercial biocontrol product based on *P. gigantea*, is commonly (>47 000 ha annually) used in forestry in the Nordic countries [4]. However, very little is known about the mechanism of the biocontrol action. Research has been focused on upregulated genes during interaction between *P. gigantea* and *Heterobasidion* spp. [5], induced resistance barriers, and metabolites accumulated by the host due to prior colonization of *P. gigantea* on stumps [4] etc., but very little of the research efforts have been focused on the impact of secondary metabolites produced by *P. gigantea* on *Heterobasidion* spp. Only 2′,3′,5′-trimethoxy-*p*-terphenyl has previously been reported to be produced by *P. gigantea* [6], but the biological activity of this compound has not been examined. However, some other *p*-terphenyls have been shown to have biological activities, e.g., antifungal, antibacterial, antioxidant, and cytotoxic [7,8]. 

The objective of the present study was to investigate the production of secondary metabolites by *P. gigantea*, and to test whether these compounds have an effect on *Heterobasidion*, and thus, could be involved in the in vivo biocontrol interactions between *P. gigantea* and *Heterobasidion* species. The paper describes the isolation and characterization of five compounds, of which three were new diarylcyclopentenones, one was a new *p*-terphenyl natural product, and one was the known compound *o*-orsellinaldehyde. Additionally, the biosynthesis of the compounds is discussed. 

## 2. Results and Discussion

### 2.1. Isolation of Compounds

A four liter liquid culture of *P. gigantea* was subjected to solid-phase extraction (SPE) resulting in a 20% CH_3_CN extract and a 95% CH_3_CN extract. Each extract was fractionated by semi-preparative RP-HPLC and fractions corresponding to major UV peaks were collected and dried, resulting in the isolation of five compounds. Compounds **1** and **2** were found in the 20% CH_3_CN extract and compounds **3** and **4**, and *o*-orsellinaldehyde, were obtained from the 95% CH_3_CN extract.

### 2.2. Structure Determination

**Figure 1:**
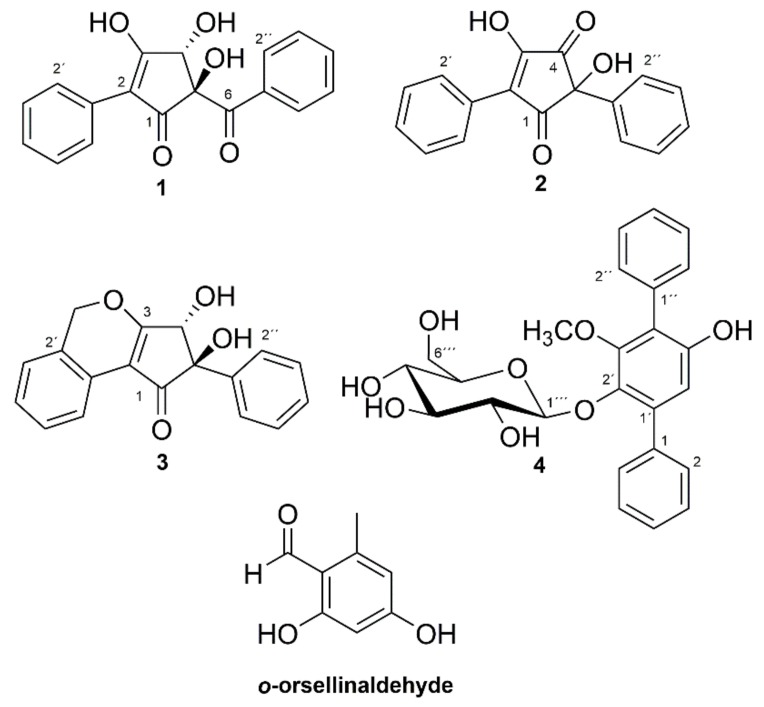
Structures of compounds **1**–**4** and *o*-orsellinaldehyde. Relative configuration shown for compounds **1** and **3**.

**Figure 2:**
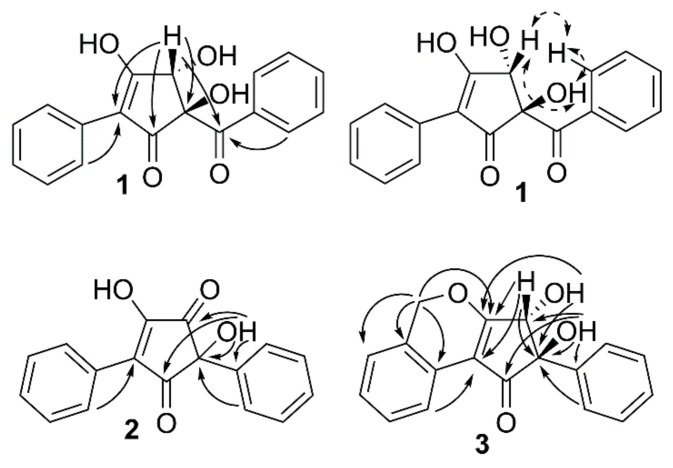
Key HMBC (solid arrows) and ROESY (dashed arrows) correlations for structure determination of compounds **1**–**3**.

The structures of the compounds (Figure 1) were elucidated using data from liquid chromatography—high-resolution mass spectrometry (LC-HRMS) and NMR spectroscopy. The supporting 1D and 2D NMR spectra and HRMS data for compounds **1**–**4** are presented in the supplementary material file. The ^1^H- and ^13^C-NMR data for compounds **1**–**3** are presented in Table 1, and for compound **4** in Table 2, while key heteronuclear multiple-bond correlations (HMBC) and rotating-frame Overhauser enhancement spectroscopy (ROESY) correlations are given in Figure 2. *o*-Orsellinaldehyde was identified by HRMS and ^1^H-NMR by comparison with literature data [9].

Compound **1** had the molecular formula C_18_H_14_O_5_, based on LC-HRMS analysis, corresponding to an unsaturation index of 12. The one-dimensional ^1^H-NMR spectrum of **1** (Table 1) in MeOH-*d*_4_ showed six signals in the aromatic region from, in total, ten hydrogens, with coupling patterns in agreement with two different non-substituted phenyl groups, and also a singlet signal at δ_H_ 4.76 from a methine group, which according to the carbon chemical shift (δ_C_ 80.8, C-4), should be oxygen linked. The two non-substituted phenyl rings contributed with eight to the calculated unsaturation index (12), leaving out four to a central moiety which must contain six carbons, four hydrogens and five oxygens. The multiplet at δ_H_ 8.01 (H-2′′/H-6′′) showed an HMBC correlation (Figure 2) to a carbon at δ_C_ 201.7 (C-6), placing a carbonyl group immediately adjacent to one of the phenyl rings. Similarly, the multiplet at δ_H_ 7.77 (H-2′/H-6′) from the second phenyl ring showed an HMBC correlation to an sp^2^ carbon next to that phenyl ring (δ_C_ 117.5, C-2). HMBC experiments further demonstrated that the isolated proton (δ_H_ 4.76, δ_C_ 80.8, H-4) was in the proximity of both C-2 and C-6. In addition to these HMBC correlations, H-4 also showed correlations to a carbon at δ_C_ 89.9 (C-5) and to a broad signal at 190.9 (C-1), probably from another carbonyl function. 

Of the remaining four unsaturations, two were taken by the indicated carbonyl groups and one by the double bond to C-2, which means that compound **1** should contain a five-membered ring placed between one phenyl ring and one benzoyl moiety to fulfill the unsaturation index. Since only one of the remaining four hydrogens gave signals in the ^1^H-NMR spectrum (MeOH-*d*_4_), compound **1** must contain three hydroxyl groups placed on the five membered ring. Of the six carbons in the central moiety, only five gave signals in the ^13^C-NMR spectrum—the signal for C-3 was missing. The broad nature of the C-1 signal and its relatively low chemical shift, plus the missing signal of C-3 could be explained by a β-hydroxylated-α,β-unsaturated keto function, which interconverts by tautomerization. Alternatively, the broad signal at 190.9 includes both the signal of C-1 and C-3. Finally, the two remaining hydroxyl groups could be placed on C-4 and C-5. In THF-*d*_8_, these two hydroxyl protons resonated at δ_H_ 6.02 and δ_H_ 5.18, 5-OH, and 4-OH, respectively, as assigned by a COSY correlation between H-4β and 4-OH. There was a ROESY correlation between H-4β and 5-OH (Figure 2). Energy minimized models of **1** with *trans* and *cis* relation between 5-OH and 4-OH were compared. In *trans*-**1**, the distance H-4β/5-OH was 2.2 Å, and the 5-OH/4-OH distance was 3.5 Å, whereas in *cis*-**1**, the distances were 3.0 Å and 2.8 Å, respectively. The presence of a ROESY correlation between H-4β and 5-OH, but not between 5-OH and 4-OH, fits the *trans* configuration better, and thus, the structure of compound **1** was concluded to be as shown in Figure 1, whereby **1** was named phlebiopsin A.

LC-HRMS analysis established the molecular formula for compound **2** to be C_17_H_12_O_4_ corresponding to an unsaturation index of 12. The ^1^H-NMR (Table 1) spectrum of compound **2** in MeOH-*d*_4_ was overall similar to the spectrum of compound **1**, although a signal equivalent to H-4 of compound **1** was missing. Signals from ten protons in the region δ_H_ 7.3–7.6, suggested that two non-substituted phenyl rings were present just as in compound **1**. Correlation spectroscopy (COSY), heteronuclear single quantum coherence (HSQC), and HMBC data were used to assign the corresponding sp^2^ carbon signals in the ^13^C spectrum. In addition to the aromatic carbons, the ^13^C spectrum contained signals from two carbonyls (δ_C_ 199.8 and 199.0), two olefinic carbons (δ_C_ 167.6 and 130.6) and one quaternary carbon (δ_C_ 77.9). These findings contributed with further three unsaturations to the eight accounted for by the phenyl rings, leaving out one (unsaturation index 12) to a five-membered ring, similar to compound **1**. HMBC (Figure 2) displayed correlations between H-2′/H-6′ and one of the olefinic carbons (δ_C_ 130.6, C-2) and between H-2′′/H-6′′ and the quaternary carbon (δ_C_ 77.9, C-5). Consequently, the remaining olefinic carbon (δ_C_ 167.6) and two carbonyls are part of the five-membered ring, and by comparing with the molecular formula, the five-membered ring should be substituted by two hydroxyl groups. The structure proposed for compound **2** is the only one that fits these data, except for the other tautomer. A ^1^H-NMR spectrum of compound **2** was also recorded in THF-*d*_8_, showing a proton signal at δ_H_ 5.99 from the hydroxyl group at C-5. Furthermore, HMBC correlations between 5-OH and C-5, C-1′′, C-4, and a weak correlation between 5-OH and C-1, further strengthened the proposed structure of compound **2**. Compound **2** was given the name phlebiopsin B. Phlebiopsin B is very similar to gyroporin [10], which has two *p*-hydroxyphenyl groups instead of two phenyl groups as in **2**.

LC-HRMS data of compound **3** established the molecular formula as C_18_H_14_O_4_, thus implying 12 unsaturations. Judged by the molecular formula, a structure with similarities to compound **1** and **2** was expected. Evidently, the ^1^H-NMR spectrum in THF-*d*_8_ of compound **3** contained several multiplets between δ_H_ 7.13 and 8.11. However, these signals integrated for nine protons and not ten as in both compound **1** and **2**, thus, suggesting an additional substituent on one of the phenyl rings. Assisted by HSQC data, one methylene (δ_H_ 5.61/5.50, d, 13.6 Hz, 2′-CH_2_), two hydroxyl groups (δ_H_ 5.45, s, 5-OH, and δ_H_ 4.61, d, 6.6 Hz, 4-OH) and one methine group (δ_H_ 4.69, d, 6.6 Hz, H-4) were identified in the ^1^H-NMR spectrum. From HMBC experiments (Figure 2), it was found that the hydroxyl group at δ_H_ 5.45 (5-OH) was in the vicinity of a carbonyl carbon (δ_C_ 198.7, C-1), an sp^2^ carbon (δ_C_ 141.6, C-1′′), a quaternary carbon (δ_C_ 86.0, C-5), and a methine group (δ_C_ 78.0, C-4). Furthermore, both H-4 and the other hydroxyl group (4-OH) displayed HMBC correlations to a carbon at δ_C_ 182.4 (C-3) and to C-5. In addition, H-4 had an HMBC correlation to a carbon at δ_C_ 114.1 (C-2). It was also observed by HMBC experiments that H-2′′/H-6′′ was within three bonds from C-5. Altogether, the HMBC data suggested this part of the molecule to be an α,β-unsaturated cyclopentenone ring linked via C-5 to a non-substituted phenyl ring and hydroxylated at C-4 and C-5, similar to compound **1**, but without the carbonyl joining the phenyl and the pentenone ring. The relation of the two hydroxyl groups were also proposed to be the same as in compound **1**, that is *trans*, based on an observed ROESY correlation between 5-OH and H-4. On the other side of the cyclopentenone ring, an HMBC correlation was observed between H-6′ and C-2 that connected the second phenyl ring with the cyclopentenone ring. HMBC data also demonstrated that a methylene bridge must be present based on correlations from the methylene at δ_H_ 5.61/5.60 (2′-CH_2_) to C-3, C-1′, C-2′, and C-3′. Thus, the last unsaturation could be attributed to another ring and fulfill the unsaturation index (12), and finally, the structure of compound **3** could be proposed. Compound **3** was given the name phlebiopsin C.

Compounds **2** and **3** are hydroxylated 2,5-diarylcyclopentenones and compound **1** has a related structure. The 2,5-diarycyclopentenones is a group of compounds that are rarely found in nature, and they have been described as characteristic constituents of certain mushrooms of the order Boletales, and responsible for the bluing reactions observed when the fungal fruit bodies are exposed to mechanical injury. Examples include gyrocyanin and gyroporin isolated from *Gyroporus cyanescens* [10] and *Chamonixia caespitosa* [11], chamonixin from *C. caespitosa* [11] and *Gyrodon lividus* [12], as well as involutone and involutin from *Paxillus involutus* [13,14,15]. However, the previous finding of the 2,5-diarylcyclopentenones sydowin A and B, in the ascomycete *Aspergillus sydowii* [16], and now the finding of the 2,5-diarylcyclopentenones **2** and **3** in *P. gigantea*, order Polyporales, which is the first finding in a basidiomycete outside the order Boletales, show that these compounds are not exclusively produced in the order Boletales only. 

Compound **4** was assigned the molecular formula C_25_H_26_O_8_, based on LC-HRMS data, corresponding to an unsaturation index of 13. The one-dimensional ^1^H-NMR spectrum in acetone-*d*_6_ contained signals from eleven protons linked to sp^2^ carbons between δ_H_ 6.7 and 7.6, a one-proton doublet CH signal at δ_H_ 4.81 (δ_C_ 104.6, C-1′′), several multiplet signals from six CH protons between δ_H_ 3.0 and 3.6, and a methoxy signal at δ_H_ 3.59 (δ_C_ 61.3, –OCH_3_). Data from COSY, TOCSY, HSQC, and HMBC experiments suggested the compound to be an analogue to terfestatin A [17], with a methoxy group on the central aromatic ring instead of a hydroxyl function. This methoxy analogue of terfestatin A has previously been synthesized, and the NMR data for **4** (Table 2) was in good agreement with literature values [18]. However, the optical rotation for compound **4** (−43°) was different from what was reported for the synthesized compound (+128.8°) [18], but was similar to what was reported for terfestatin A (−37.2°) [17]. To avoid any ambiguities concerning absolute configuration, the glucose residue was analyzed by a diastereomeric resolution method, which clearly indicated that compound **4** had a d-glucopyranosyl residue. This is the first report on compound **4** as a naturally occurring compound, and the fully assigned NMR data is presented here for the first time (Table 2). Compound **4** was given the name methyl-terfestatin A. One *p*-terphenyl compound, 2′,3′,5′-trimethoxy-*p*-terphenyl, has previously been reported from *P. gigantea* [6], but this compound was not found in the present study. 

**Figure 3:**
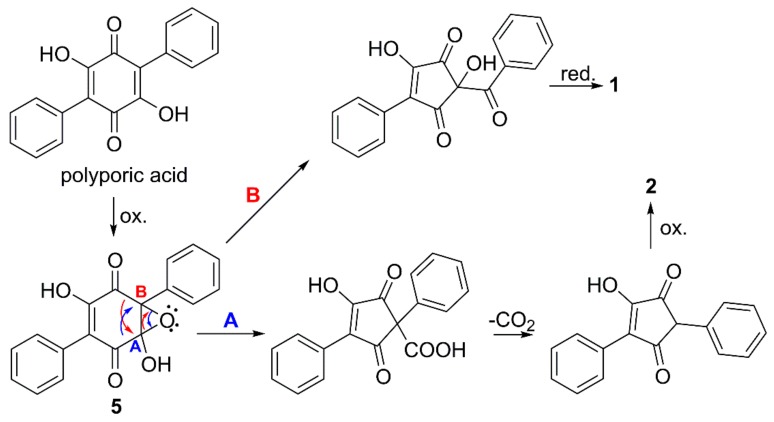
Proposed formation of compounds **1** and **2** from polyporic acid, via the monooxygenase generated intermediate **5**, and followed by two alternate openings of the epoxide (A and B).

Isotope labelling studies suggested more than 50 years ago the biosynthesis of *p*-terphenylquinones, such as polyporic acid and atromentin, to proceed by condensation of two molecules of phenylpyruvic acid or similar [19]. Natural 2,5-diarylcyclopentenones, e.g., gyrocyanin, have been proposed to be formed by oxidative ring contraction of atromentin [10], but only recently, genetic, transcriptomic, and chemical evidence were presented, showing that atromentin, indeed, was the direct precursor of gyrocyanin in *P. involutus* (Boletales) [20]. It is possible that the cyclopentenone motifs of compounds **2** and **3** are formed in a similar way, but from polyporic acid via the putative monooxygenase generated epoxide **5** (Figure 3, route A). The cyclopentenone motif of compound **1** may originate from an alternate opening of epoxide **5** (Figure 3, route B), which would leave a keto function between the cyclopentenone group and one of the phenyl groups.

As mentioned above, there were difficulties in observing sharp ^13^C-NMR signals for carbon 1 and 3 in compound **1**, even though several different solvents and temperatures were tested (data not shown). By contrast, compound **2** and **3** showed excellent signals for the corresponding carbon atoms. An explanation could be that compound **1** interconverts by keto–enol tautomerization, whereas compound **2** and **3** are locked; compound **2** by intramolecular hydrogen bonding to the keto function at C-4, and compound **3** by the ether. Gyroporin, involutin, chamonixin, and sydowin A and B are compounds that show high degree of structural similarities with compound **1**–**3**. Their structures do all consist of a β-hydroxylated-α,β-unsaturated keto function, and thus, could be associated with tautomerism. Evidently, difficulties and/or notable chemical shifts have been observed for the signals of C-1 and C-3 for these structures. In sydowin A, for example, the chemical shifts for C-1 and C-3 were reported as 194.9 and 194.1, respectively, and the corresponding carbon atoms in sydowin B were never detected [16]. Furthermore, Feling et al. [21], were able to detect C-1 and C-3 in chamonixin, but not in involutin, even though the structures of these compounds only differ in a hydroxyl group in one of the phenyl rings. Involutin was also identified by Antkowiak et al. [15], but in contrast to Feling et al. [21], they were able to observe ^13^C signals for C-1 and C-3. In conclusion, these type of structures, with the inherent possibility of keto–enol tautomerization, seem to result in various levels of difficulty during the NMR analysis. The exact behavior of the compounds during analysis probably depends on factors such as solvent, pH, and temperature. In the case of compound **1**, many different NMR solvents and temperatures were tested with unsatisfactory results.

### 2.3. Biological Activities

Compounds **1**–**4** and *o*-orsellinaldehyde were tested against three species of fungi, including the basidiomycete *H. occidentale* and the ascomycetes *F. oxysporum* and *P. canescens*, for determination of minimal inhibitory concentration (MIC). *Heterobasidion occidentale* is a common pathogen of conifers, causing extensive economic losses. The other two fungal species represent model organisms, among which *F. oxysporum* is a common pathogen of many plant species, while *P. canescens* is a saprotroph. Compounds **1**–**4** were tested in concentrations from 400 μM down to 1 nM, whereas *o*-orsellinaldehyde was tested from 2 mM down to 1 nM. However, none of compounds **1**–**4** were able to inhibit the germination of fungal conidia at the tested concentrations. As mentioned above, compound **4** is very similar to terfestatin A, which has been characterized as a specific inhibitor of auxin signaling in plants [17,22]. In a subsequent study, aimed at identifying the active core structure of terfestatin A, compound **4** was synthesized and also tested for inhibition of auxin signaling, resulting in the conclusion that the hydroxyl group at position C-3′ is critical for inhibition [18]. Gliocladin B, also identified as a *p*-terphenyl β-glucoside, has been shown to inhibit the growth of two human tumor cell lines (HeLa and HCT 116), as well as having antimicrobial activity [23]. It differs in structure from compound **4** by having methoxy groups at C-4 and C-4′′, and by a hydroxyl group at 3′, as terfestatin A, instead of a methoxy group.

In contrast to compounds **1**–**4**, *o*-orsellinaldehyde exhibited complete inhibition of conidial germination of *H. occidentale* down to 1 mM, and partial inhibition at 0.5 mM. Furthermore, *o*-orsellinaldehyde showed complete inhibition of conidial germination of both *P. canescens* and *F. oxysporum* at 2 mM and partial inhibition down to 1 mM. *o*-Orsellinaldehyde has never, as far as we know, been investigated regarding its antifungal activity, but it has been shown to be cytotoxic against a human carcinoma cell line (Hep 3B) and a lung fibroblast cell line (MRC-5) [24].

The mode of action of *P. gigantea* in biocontrol of *Heterobasidion* spp. has previously been suggested to be by resource competition [5] as the biocontrol agent is able to rapidly colonize the surfaces of fresh tree stumps, thereby making the niche suitable for *Heterobasidion* infections pre-occupied. The present study, with no antifungal activity found for compounds **1**–**4**, and rather weak activity for *o*-orsellinaldehyde, supports the suggested mode of action.

## 3. Materials and Methods

### 3.1. General Experimental Procedures

Optical rotations were measured in CH_3_OH on Perkin-Elmer polarimeters models 341 and 343, using a 1 mL cell (path length 10.0 cm, λ 589 nm, 20 °C). UV spectra were recorded in CH_3_OH on a Hitachi U-2001 spectrophotometer. NMR spectra were recorded with Bruker Avance III 600 MHz spectrometers (Bruker Biospin GmBH, Rheinstetten, Germany), equipped with a 5 mm ^1^H/^13^C/^15^N/^31^P inverse detection CryoProbe or a 5 mm broadband observe detection SmartProbe, operated at 600 MHz for ^1^H-NMR and 150 MHz for ^13^C-NMR. NMR experiments were performed with MeOH-*d*_4_, acetone-*d*_6_, or THF-*d*_8_ as solvents, using pulse sequences supplied by Bruker to perform 1D ^1^H and ^13^C, and 2D ^1^H–^1^H COSY, ^1^H–^1^H TOCSY (80 ms), ^1^H–^13^C HSQC-DEPT, ^1^H–^13^C HMBC (65 ms), and ^1^H–^1^H ROESY (200 or 300 ms) experiments. Chemical shifts are reported relative to residual solvent signals (δ_H_ 3.31; δ_C_ 49.15 for MeOH-*d*_3_, δ_H_ 2.05; δ_C_ 29.92 for acetone-*d*_5_ and δ_H_ 3.58; δ_C_ 67.57 for THF-*d*_7_). LC-HRMS was performed on an HP1100 LC system (Hewlett-Packard, Palo Alto, CA, USA)) using a Reprosil-Pur ODS-3 column (C18, 5 μm, 4 × 125 mm, Dr. Maisch GmbH, Ammerbuch, Germany) connected to a Bruker maXis Impact mass spectrometer (Bruker Daltonic GmbH, Bremen, Germany) with an electrospray ion-source (ESI-QTOF). Calibration was performed prior to analysis with a sodium formate solution, and additionally, internally for each run by injecting the calibrant at the beginning of each run using a software controlled valve. Semi-preparative HPLC was run using a Gilson system, including a Gilson 305/306 pump, a Gilson 118 UV detector (λ 254 nm) and a Gilson FC204 fraction collector (Gilson Inc., Middleton, WI, USA).

### 3.2. Production of Fungal Cultures for Metabolite Isolation

*P. gigantea* strain Rotstop^®^ (Verdera Ltd., Esbo, Finland) was maintained on Melin Norkrans agar media (MMN; [25]) in 9 cm diameter Petri dishes at room temperature (ca. 21 °C) in the dark. For the production of metabolites, 500 mL Erlenmeyer flasks were used, each containing 250 mL of liquid MMN media. Five agar plugs 0.5 × 0.5 cm in size with established fungal mycelia from an actively growing colony were aseptically inoculated in each flask, and incubated on a rotary shaker at 120 rpm at room temperature for seven weeks. Cultures were filtered to obtain a mycelium-free sample for sample work-up by reversed-phase SPE.

### 3.3. Production of Microbial Inoculums for Bioassay

The fungal isolates *H. occidentale* strain TC122-12, *F. oxysporum* strain 4S4-49b, and *P. canescens* strain 9P2-24a were obtained from the culture collection of the Department of Forest Mycology and Plant Pathology, Swedish University of Agricultural Sciences. The isolates were cultivated on MMN in 9 cm Petri dishes at room temperature (ca. 21 °C) in the dark. Fungal conidia were harvested from 15 day old cultures by flooding the mycelium with liquid MMN media, and gently rubbing the culture colony with a glass rod. Mycelial fragments were subsequently removed from the suspensions by filtration (50 µm mesh), and the conidial concentrations were determined using a haemacytometer. 

### 3.4. Bioassay Procedure

Pure compounds **1**–**4** and *o*-orsellinaldehyde were dissolved in DMSO and transferred to wells in microtiter plates. Bioassays were performed using 100 µL of liquid MMN media containing 10^5^ conidia cells/mL of either *H. occidentale*, *F. oxysporum*, or *P. canescens* by adding respective suspensions to each well. The final concentration of DMSO was 1%. Negative control wells comprised conidia of each organism in liquid MMN media, and wells containing 10% DMSO were used as a positive control. As conidia of *P. canescens* and *F. oxysporum* germinate faster than conidia of *H. occidentale*, conidial germination for *P. canescens* and *F. oxysporum* was recorded after 65 h and for *H. occidentale* after 96 h of their inoculation and incubation at room temperature in darkness. The conidial germination and fungal growth was estimated by ocular observation of the microtiter plates using stereomicroscope, as well as by an automated plate reader (Labsystems Multiscan RC, Helsinki, Finland) operated at 620 nm. All biotests were performed in triplicate and repeated once. 

### 3.5. Extraction and Isolation

A four liter mycelium-free fungal culture of *P. gigantea* was extracted using eight 10 g Isolute C18 end-capped SPE columns. Prior to sample loading, the columns were activated with CH_3_OH (60 mL) and equilibrated with H_2_O (60 mL). The loaded columns were rinsed with 60 mL H_2_O to remove any non-bound substances before the lipophilic analytes were eluted in two steps, starting with 60 mL 20% aqueous CH_3_CN (eluate 1) followed by 95% aqueous CH_3_CN (eluate 2). The CH_3_CN of the two eluates were evaporated under reduced pressure, and the residues lyophilized, yielding 320 mg dry material of eluate 1 and 70 mg of eluate 2. The dry SPE extracts were dissolved in aqueous CH_3_CN (15% and 30% CH_3_CN for eluate 1 and 2, respectively) and subjected (4 × 1 mL injected of each eluate) to semipreparative reversed phase HPLC (Reprosil-Pur ODS-3, 5 μm, C18, 20 × 100 mm, with a guard column, 20 × 30 mm, 5 μm, Dr. Maisch GmbH). The column was eluted at 13.2 mL/min with aqueous CH_3_CN (0.2% formic acid), using the gradient 8–70% CH_3_CN in 50 min, 70–95% CH_3_CN in 5 min, and a hold at 95% for 5 min. The elution was monitored at 254 nm and fractions were collected in 2 mL 96-deep well plates. Fractions corresponding to major UV peaks were selected, pooled separately, and concentrated gently by nitrogen gas, and diluted 1:1 with H_2_O prior to small scale SPE extraction (Waters Oasis, HLB, 30 mg, 1 mL). The columns were activated (1 mL CH_3_OH) and equilibrated (1 mL H_2_O) before sample loading. Following sample loading, H_2_O was replaced by rinsing the SPE columns with D_2_O. The columns were subsequently eluted with either MeOH-*d*_4_ (compounds **1**–**2**) or chloroform-*d* (compounds **3**–**4**) directly into 5 mm NMR tubes. When necessary, NMR solvents were changed to acetone-*d*_6_ or THF-*d*_8_. LC-HRMS data were recorded using the same gradient and mobile phases as for the preparative system at 0.7 mL/min. Compounds **1** and **2** were isolated from the 20% CH_3_CN extract, whereas compounds **3** and **4** were isolated from the 95% CH_3_CN extract.

### 3.6. Relative Configuration of Compound ***1***

Energy minimized models of compound **1** were calculated by using Chem3D Ultra ver. 16.0.1.4, with the MM2 force field (PerkinElmer Informatics Inc., Waltham, MA, USA). Subsequently, the relevant interatomic distances were measured using the same software.

### 3.7. Absolute Configuration of Compound ***4***

Approximately 90 μg of compound **4** in MeOH (50 μL) was treated with 500 μL MeOH–acetyl chloride (10:1), and heated at 85 °C overnight, after which the mixture was dried by nitrogen gas. The residue was redissolved in 500 μL (*S*)-2-butanol-acetylchloride (10:1), and heated at 85 °C for 3 h. Reference samples consisting of methyl α-d-glucopyranoside, treated with both (*S*)-2-butanol and racemic 2-butanol, were prepared in the same manner. The resulting 2-butyl glycosides were dried by a stream of nitrogen gas, and subsequently silylated by adding 100 μL pyridine and 100 μL BSTFA–TMSCl, 99:1 (Sylon BFT, Supelco). The solutions were kept at room temperature overnight. Following dilution with ethyl acetate (1 mL) all samples were analyzed by GC-MS using a fused silica column (HP-5MS; 0.25 µm, 30 m × 0.25 mm, Agilent) with a temperature gradient (80 °C for 5 min, 80–250 °C at 5 °C/min, and a final hold at 250 °C for 5 min). The GC injector was held at 240 °C, and the GC-MS interface at 240 °C. Samples (1 µL) were injected in split mode (50:1), and helium was used as carrier gas (1 mL/min). This derivatization procedure results in a mixture of mainly 2-butyl α/β-glucopyranosides and the (*S*)-2-butyl and (*R*)-2-butyl glycosides of one of these anomeric forms are well separated by GC [26]. The (*S*)-2-butyl d-glucopyranoside yielded one GC peak at t_R_ 32.82 min and the (*R*)-2-butyl d-glucopyranoside at t_R_ 33.08. The peak of the (*R*)-2-butyl d-glucopyranoside is chromatographically equivalent to the enantiomeric (*S*)-2-butyl l-glucopyranoside, representing a standard for identification of l-glucose. 

The GC analysis of the (*S*)-2-butyl glycoside of compound **4** resulted in one major peak at t_R_ 32.85 min and one minor peak at t_R_ 33.11 min (ratio approx. 10:1, diastereomeric compound formed from contaminating (*R*)-2-butanol in the reagent), identifying the monosaccharide unit of compound **4** as d-glucose. 

### 3.8. Phlebiopsin A *(**1**)*

Compound **1** was obtained as a yellowish oil; 0.36 mg; [α]${}_{D}^{20}$ +33.8 (*c* 0.04, CH_3_OH); UV (CH_3_OH) λ_max_(log ε) 381 (2.3), 274 (4.2), 203 (4.4) nm; ^1^H- and ^13^C-NMR in MeOH-*d*_4_, see Table 1; HRMS *m/z* 311.0915 [M + H]^+^ (calcd for C_18_H_15_O_5_, 311.0914).

### 3.9. Phlebiopsin B *(**2**)*

Compound **2** was obtained as a yellowish oil; 1.03 mg; [α]${}_{D}^{20}$ −6.4 (*c* 0.09, CH_3_OH); UV (CH_3_OH) λ_max_(log ε) 353 (3.9), 273 (4.0), 246 (4.0), 203 (4.3) nm; ^1^H- and ^13^C-NMR in MeOH-*d*_4_, see Table 1; HRMS *m/z* 281.0808 [M + H]^+^ (calcd for C_17_H_13_O_4_, 281.0808).

### 3.10. Phlebiopsin C *(**3**)*

Compound **3** was obtained as a yellow-brownish oil; 1.29 mg; [α]${}_{D}^{20}$ −3.4 (*c* 0.12, CH_3_OH); UV (CH_3_OH) λ_max_(log ε) 288 (9.6), 254 (10.0), 205 (10.4) nm; ^1^H- and ^13^C-NMR in THF-*d*_8_, see Table 1; HRMS *m/z* 295.0970 [M + H]^+^ (calcd for C_18_H_15_O_4_, 295.0965).

### 3.11. Methyl-Terfestatin A *(**4**)*

Compound **4** was obtained as a yellow-brownish oil; 3.18 mg; [α]${}_{D}^{20}$ −43 (*c* 0.16, CH_3_OH); ^1^H- and ^13^C-NMR in acetone-*d*_6_, see Table 2; HRMS *m/z* 477.1521 [M + Na]^+^ (calcd for C_25_H_26_NaO_8_, 477.1520).

## Figures and Tables

**Table 1:** ^1^H- and ^13^C-NMR data (600 and 150 MHz, respectively) for compounds **1**–**3** at 30 °C (**1**–**2** in MeOH-*d*_4_, and **3** in THF-*d*_8_).

	1		2		3	
pos.	*δ*_C_, mult.	*δ*_H_ (J in Hz)	*δ*_C_, mult.	*δ*_H_ (J in Hz)	*δ*_C_, mult.	*δ*_H_ (J in Hz)
1	^a^ 190.9, C	-	^b^ 199.8, C	-	198.7, C	
2	117.5, C	-	^c^ 130.6, C	-	114.1, C	
3	^d^ n.d.	-	167.6, C	-	182.4, C	-
4	80.8, CH	4.76, s	^b^ 199.0, C	-	78.0, CH	4.69, d (6.6)
5	89.9, C	-	77.9, C	-	86.0, C	-
6	201.7, C	-	-	-	-	-
1′	131.6, C	-	^c^ 130.37, C	-	127.1, C	-
2′	129.3, CH	7.77, d (7.7)	130.41, CH	8.14, d (7.7)	127.7, C	-
3′	129.1, CH	7.37, t (7.7)	129.5, CH	7.47, t (7.6)	125.0, CH	7.13, d (7.5)
4′	128.4, CH	7.27, t (7.7)	130.7, CH	7.41, t (7.5)	128.9, CH	7.24, obsc.
5′	129.1, CH	7.37, t (7.7)	129.5, CH	7.47, t (7.6)	129.3, CH	7.28, t (7.5)
6′	129.3, CH	7.77, d (7.7)	130.41, CH	8.14, d (7.7)	123.3, CH	8.11, d (7.5)
1′′	138.1, C	-	138.8, C	-	141.6, C	-
2′′	131.0, CH	8.01, d (7.8)	127.1, CH	7.45, d (7.6)	128.6, CH	7.42, d (7.7)
3′′	129.0, CH	7.43, t (7.8)	129.8, CH	7.37, t (7.5)	128.0, CH	7.23, obsc.
4′′	133.7, CH	7.54, t (7.8)	129.7, CH	7.33, t (7.5)	127.72, CH	7.17, t (7.3)
5′′	129.0, CH	7.43, t (7.8)	129.8, CH	7.37, t (7.5)	128.0, CH	7.23, obsc.
6′′	131.0, CH	8.01, d (7.8)	127.1, CH	7.45, d (7.6)	128.6, CH	7.42, d (7.7)
2′-CH_2_	-	-	-	-	72.6, CH_2_	5.61, d (13.6)
						5.50, d (13.6)
5-OH		^e^ 6.02, br s		^e^ 5.99, br s		5.45, s
4-OH		^e^ 5.18, br s				4.61, d (6.6)

^a^ Broad signal, could include resonances for both C-1 and C-3; ^b^ May be interchanged; ^c^ May be interchanged; ^d^ Not detected, possibly overlapping with C-1; ^e^ Obtained in THF-*d*_8_.

**Table 2:** ^1^H- and ^13^C-NMR data (600 and 150 MHz, respectively) for compound **4** at 30 °C in acetone-*d*_6_.

pos.	*δ*_C_, mult.	*δ*_H_ (J in Hz)	pos.	*δ*_C_, mult.	*δ*_H_ (J in Hz)
1	140.0, C		5′′	128.4, CH	7.43, t (7.5)
2	130.3, CH	7.62, d (7.5)	6′′	131.5, CH	7.46, d (7.5)
3	128.4, CH	7.40, t (7.5)	1′′′	104.6, CH	4.81, d (7.7)
4	127.6, CH	7.32, t (7.5)	2′′′	75.3, CH	3.24, obsc.
5	128.4, CH	7.40, t (7.5)	3′′′	77.5, CH	3.33, td (8.8, 3.3)
6	130.3, CH	7.62, d (7.5)	4′′′	71.0, CH	3.24, obsc.
1′	137.3, C		5′′′	76.9, CH	3.05 , ddd (9.5, 4.7, 3.1)
2′	140.7, C		6′′′	62.3, CH_2_	3.53 , ddd (11.4, 5.8, 3.1)
3′	152.3, C				3.43, ddd (11.4, 7.3, 4.7)
4′	124.0, C		3′-OCH_3_	61.3, CH_3_	3.59, s
5′	151.8, C		5′-OH		7.94, s
6′	112.9, CH	6.76, s	2′′′-OH		4.10, obsc.
1′′	135.0, C		3′′′-OH		4.10, obsc.
2′′	131.5, CH	7.46, d (7.5)	4′′′-OH		4.04, d (3.3)
3′′	128.4, CH	7.43, t (7.5)	6′′′-OH		2.19, dd (7.3, 5.8)
4′′	127.6, CH	7.34, t (7.5)			

## Supplementary Materials

The following are available online. … 1D and 2D NMR spectra and HRMS data for compounds **1**–**4**.
