# Supplementary material for: Secondary Metabolites from the Root Rot Biocontrol Fungus Phlebiopsis gigantea
Source: Molecules. 2018 Jun 12;23(6):1417. doi: 10.3390/molecules23061417 (PMC6100525; doi:10.3390/molecules23061417)

Supplementary data

Secondary metabolites from the root-rot biocontrol fungus *Phlebiopsis gigantea*

David Kälvö,[†] Audrius Menkis,[‡] Anders Broberg[†*]

[†]Department of Molecular Sciences, Uppsala BioCenter, Swedish University of Agricultural Sciences, P.O. Box 7015, SE-75007, Uppsala, Sweden.

[‡]Department of Forest Mycology and Plant Pathology, Uppsala BioCenter, Swedish University of Agricultural Sciences, P.O. Box 7026 , SE-75007, Uppsala, Sweden.

[*]To whom correspondence should be addressed. Phone: +46 18 672217.

E-mail: Anders.Broberg@slu.se.

Table of contents

Phlebiopsin A **(1)**	^{1}H NMR (methanol-*d4)*	Page S3
	^{13}C NMR (methanol-*d4)*	Page S3
	COSY (methanol-*d4)*	Page S4
	HSQC (methanol-*d4)*	Page S4
	HMBC (methanol-*d4)*	Page S5
	^{1}H NMR (THF-*d8)*	Page S6
	ROESY (THF-*d8)*	Page S6
	HRMS	Page S7
Phlebiopsin B **(2)**	^{1}H NMR (methanol-*d4)*	Page S8
	^{13}C NMR (methanol-*d4)*	Page S8
	COSY (methanol-*d4)*	Page S9
	HSQC (methanol-*d4)*	Page S9
	^{1}H NMR (THF-*d8)*	Page S10
	HMBC (THF-*d8)*	Page S10
	HRMS	Page S11
Phlebiopsin C **(3)**	^{1}H NMR (THF-*d8)*	Page S12
	^{13}C NMR (THF-*d8)*	Page S12
	COSY (THF-*d4)*	Page S13
	HSQC (THF-*d4)*	Page S13
	HMBC (THF-*d8)*	Page S14
	ROESY (THF-*d8)*	Page S14
	HRMS	Page S15
Methyl-terfestatin A **(4)**	^{1}H NMR (acetone-*d6*)	Page S16
	^{13}C NMR (methanol-*d4*)	Page S16
	COSY (acetone-*d6*)	Page S17
	HSQC (acetone-*d6*)	Page S17
	HMBC (acetone-*d6*)	Page S18
	ROESY (acetone-*d6*)	Page S18
	HRMS	Page S19

^{1}H (top) and ^{13}C (bottom) NMR spectra for compound **1** (methanol-*d*$_4$, 30°C)

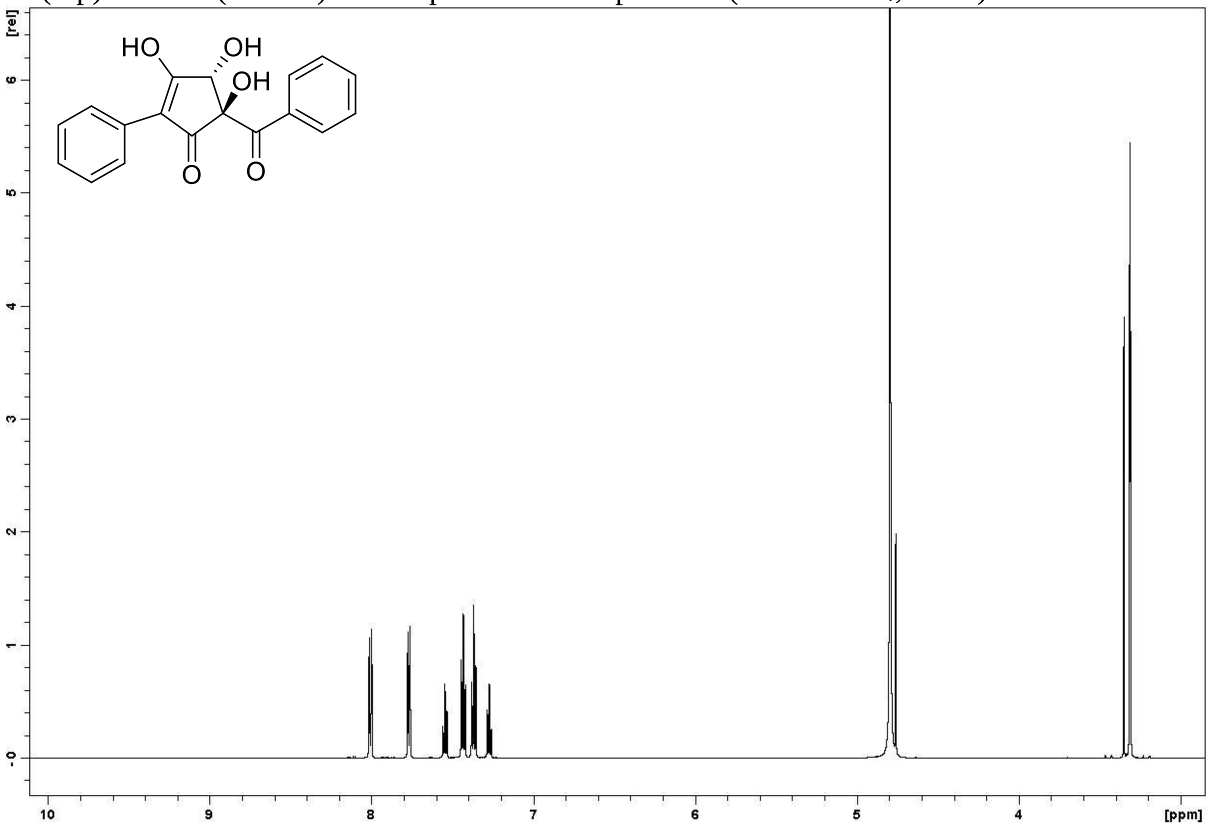

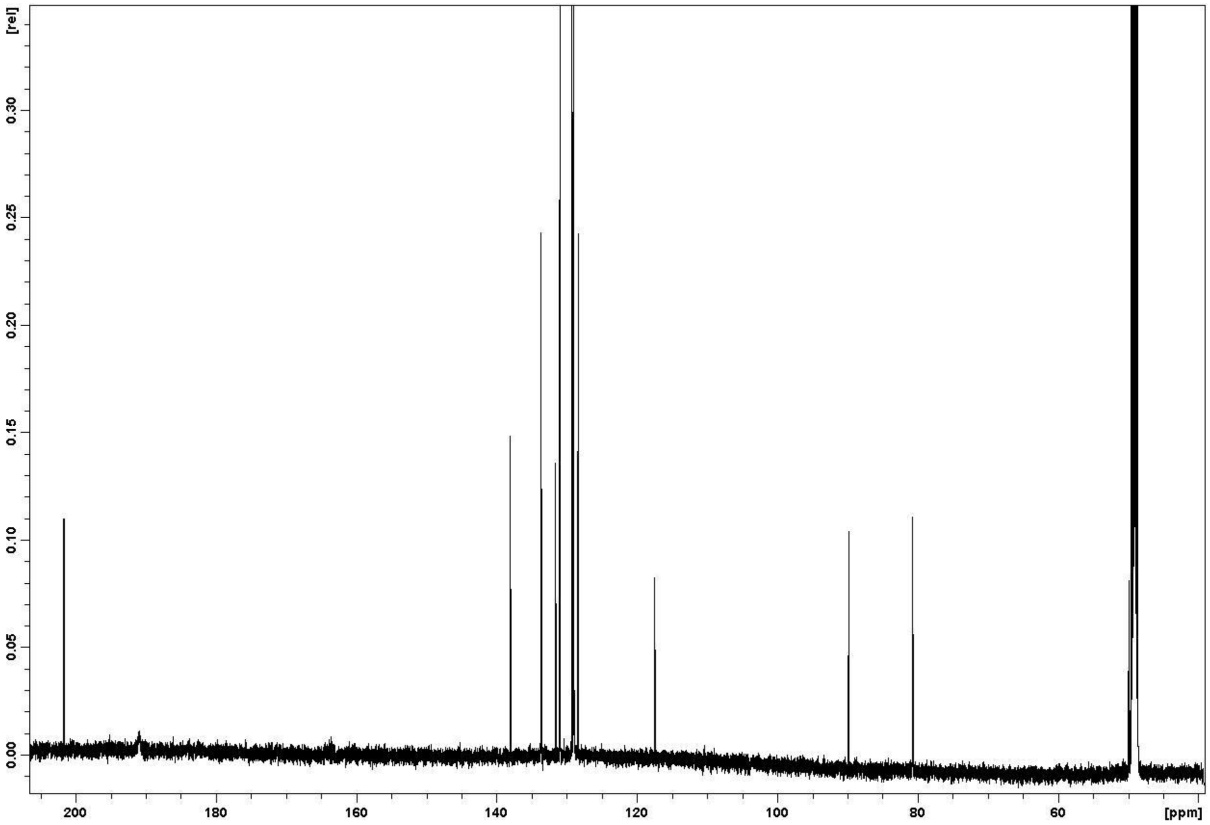

COSY (top) and HSQC (bottom) NMR spectra for compound **1** (methanol-d_4, 30°C)

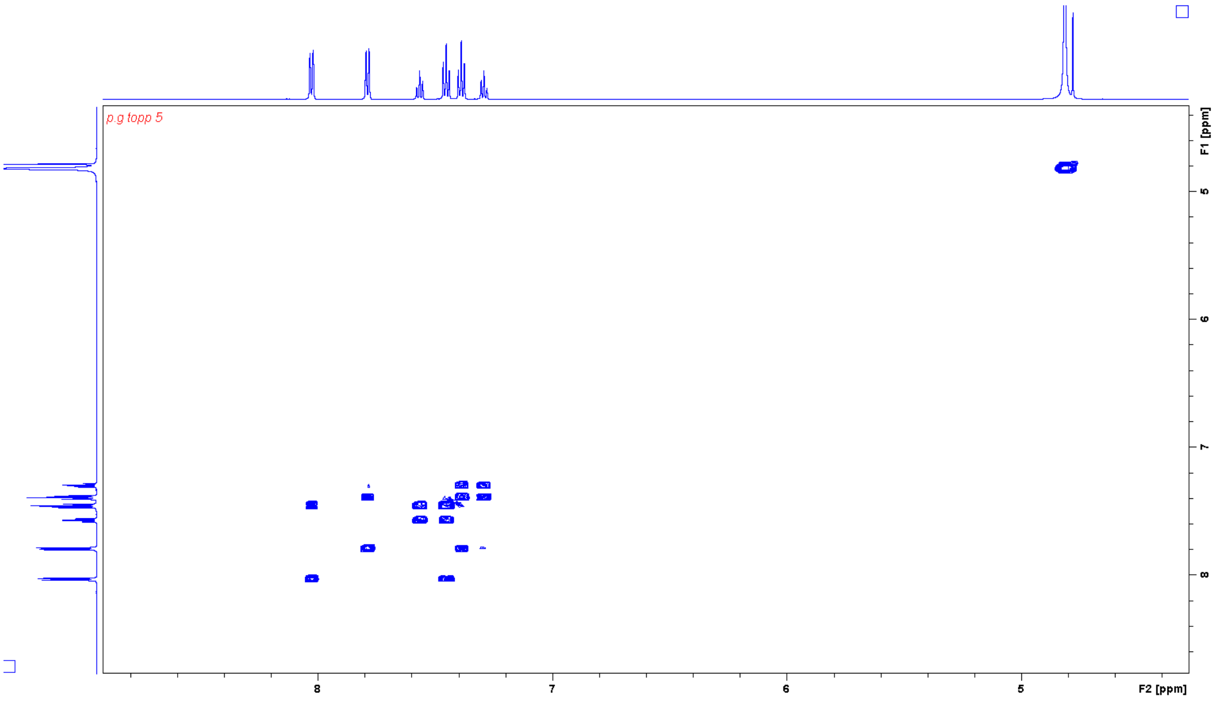

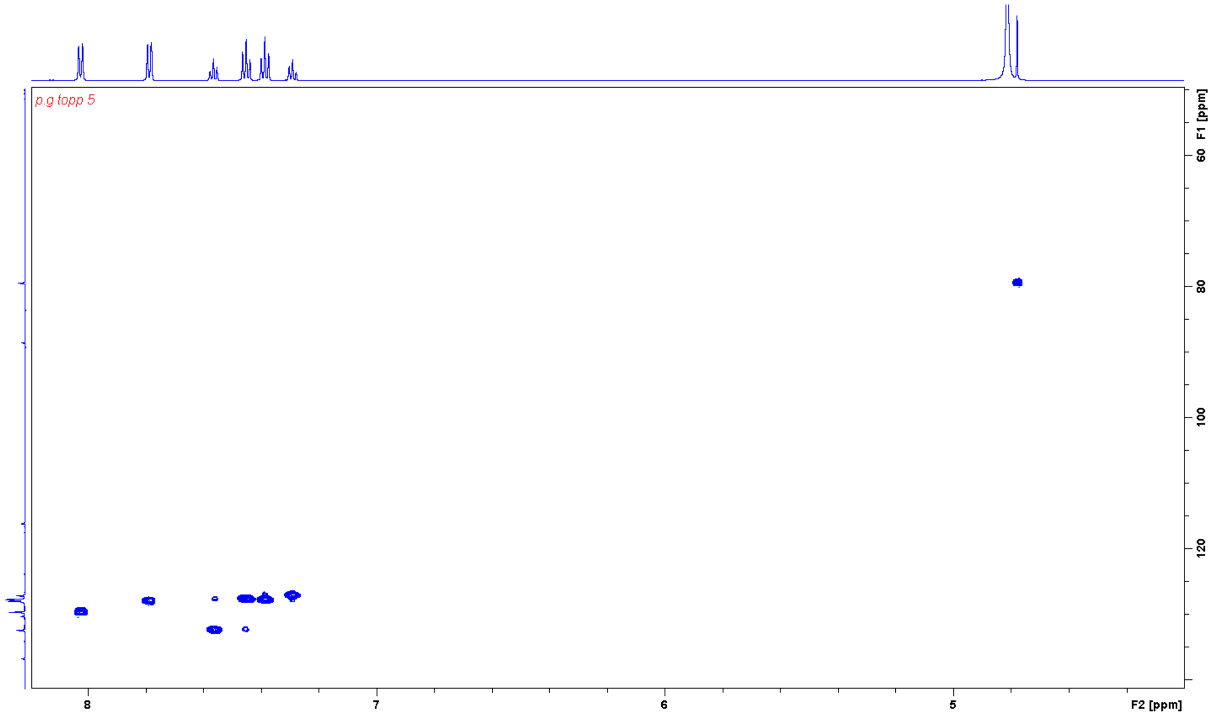

HMBC NMR spectrum for compound **1** (methanol-d_4, 30°C).

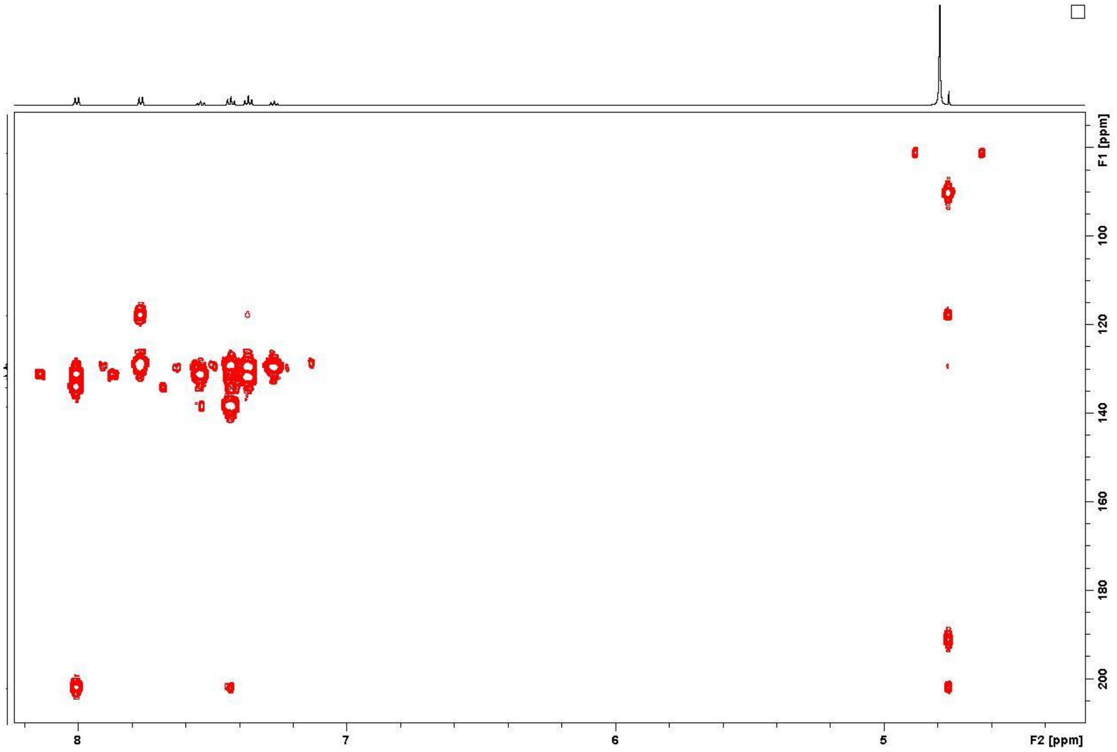

^{1}H (top) and ROESY (bottom) NMR spectra for compound **1** (THF-d_8, 30°C)

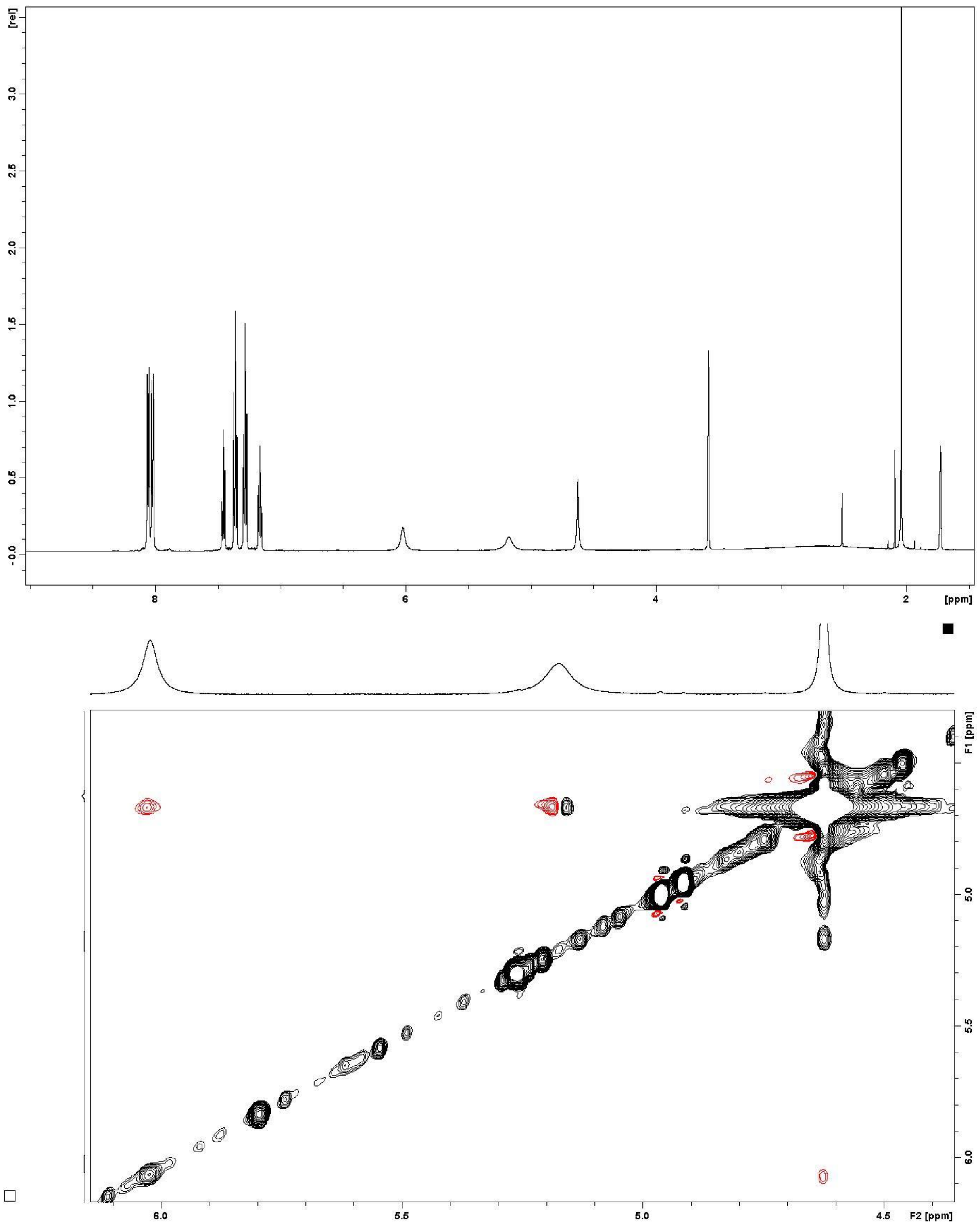

Mass spectrum for compound **1**. Major ions (from left to right): $[M+H-H_2O]^+$, $[M+H]^+$, and $[2M+Na]^+$.

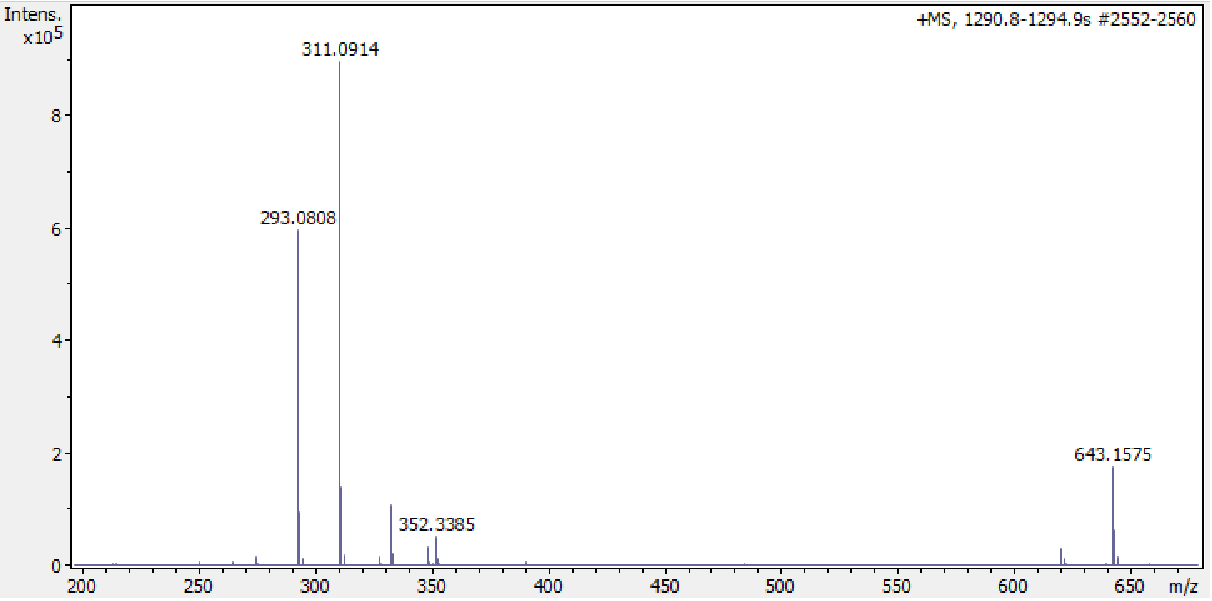

¹H (top) and ¹³C (bottom) NMR spectra for compound **2** (methanol-d_4, 30°C)

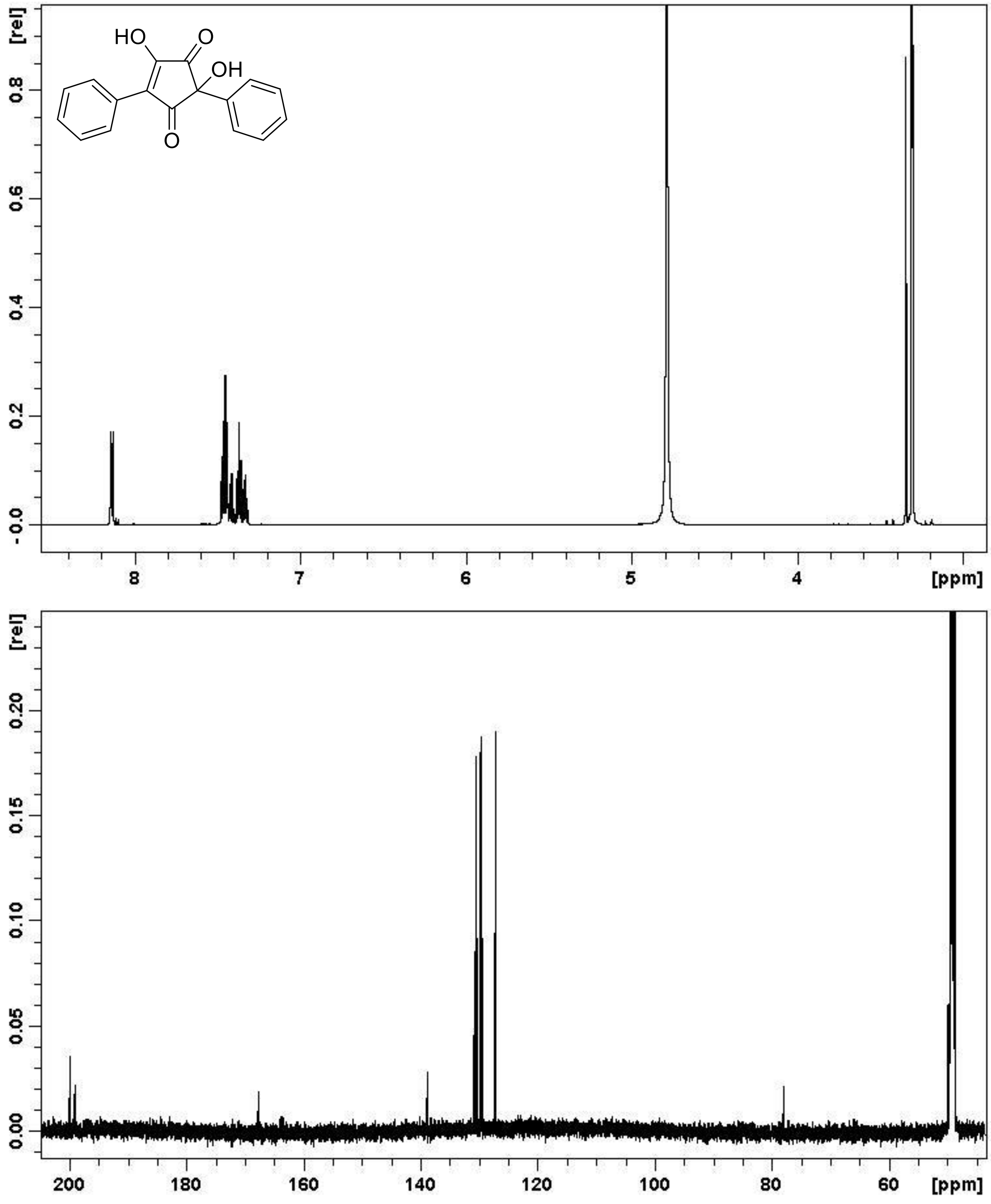

COSY (top) and HSQC (bottom) NMR spectra for compound **2** (methanol-d_4, 30°C)

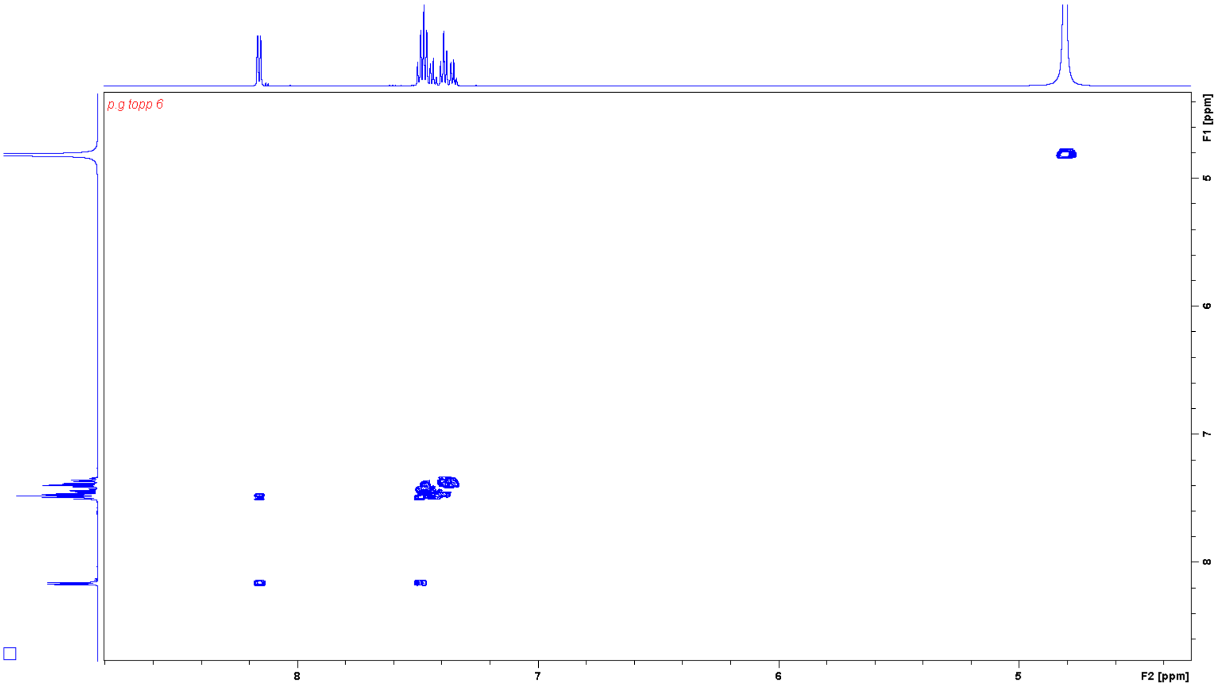

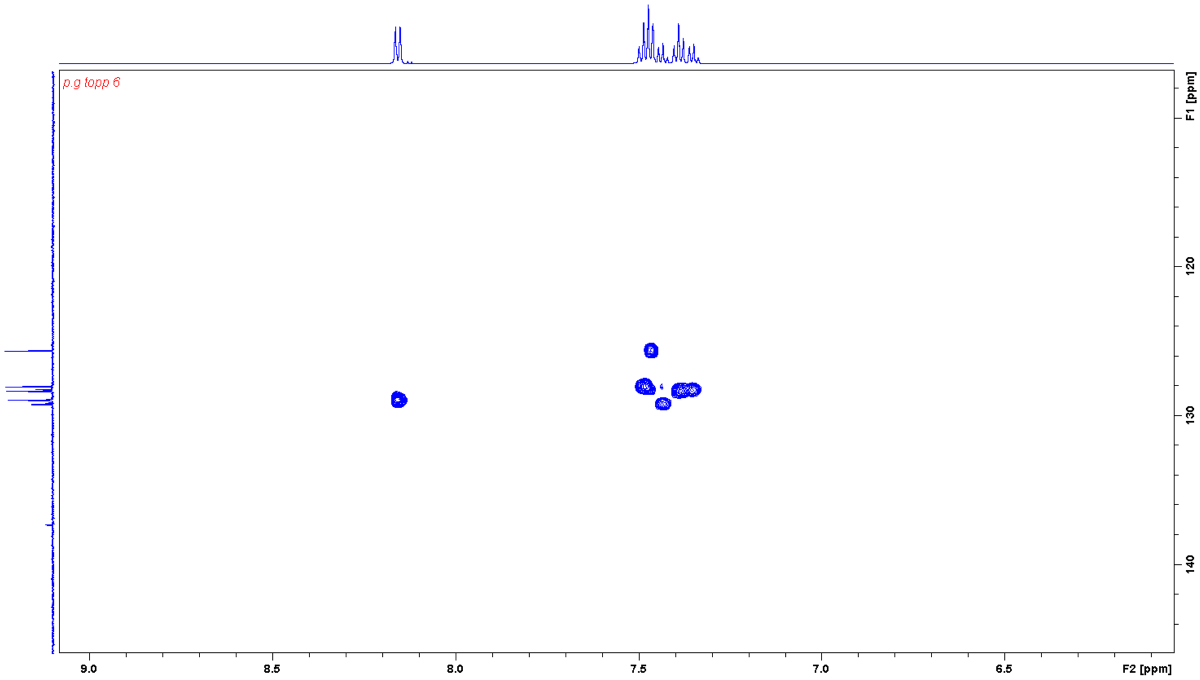

^{1}H (top) and HMBC (bottom) NMR spectra for compound **2** (THF-d_8, 30°C)

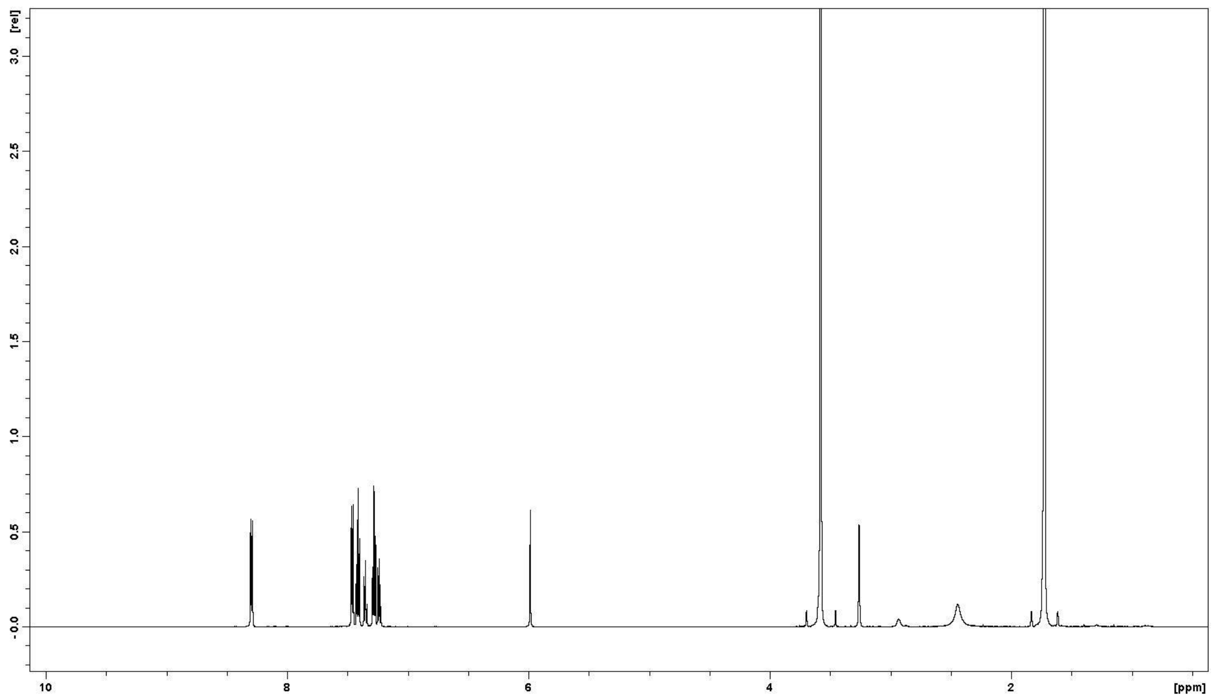

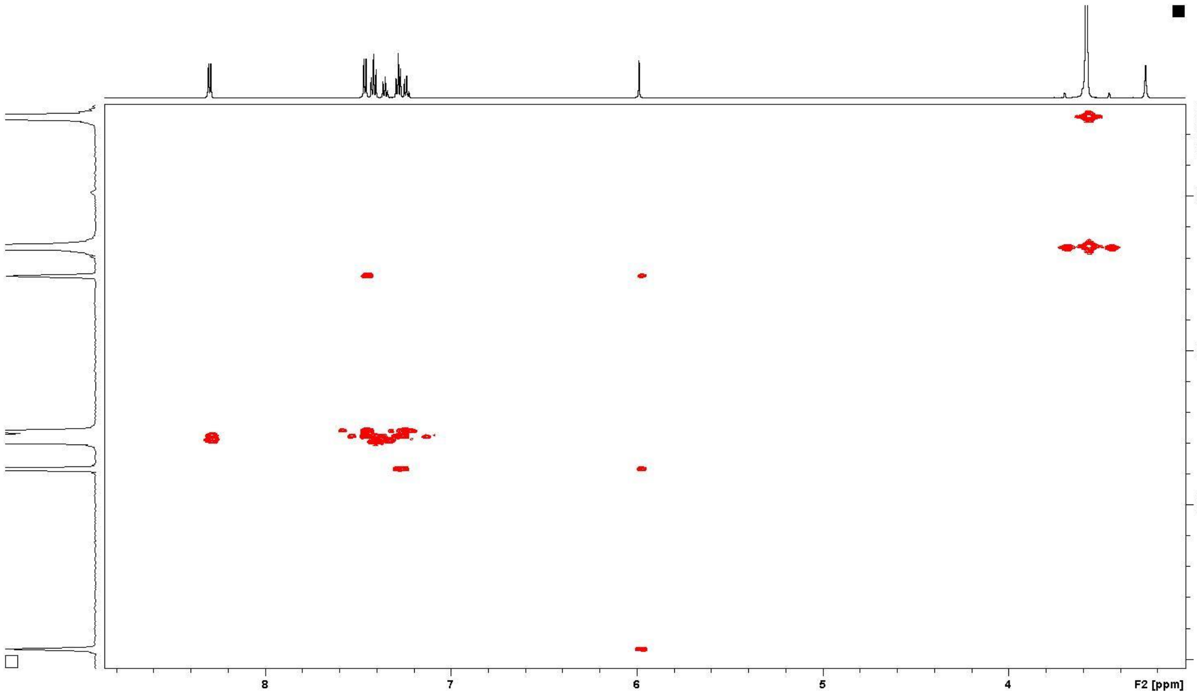

Mass spectrum for compound **2**. Major ions (from left to right): $[M+H-H_2O]^+$, $[M+H]^+$, $[M+Na]^+$and $[2M+Na]^+$.

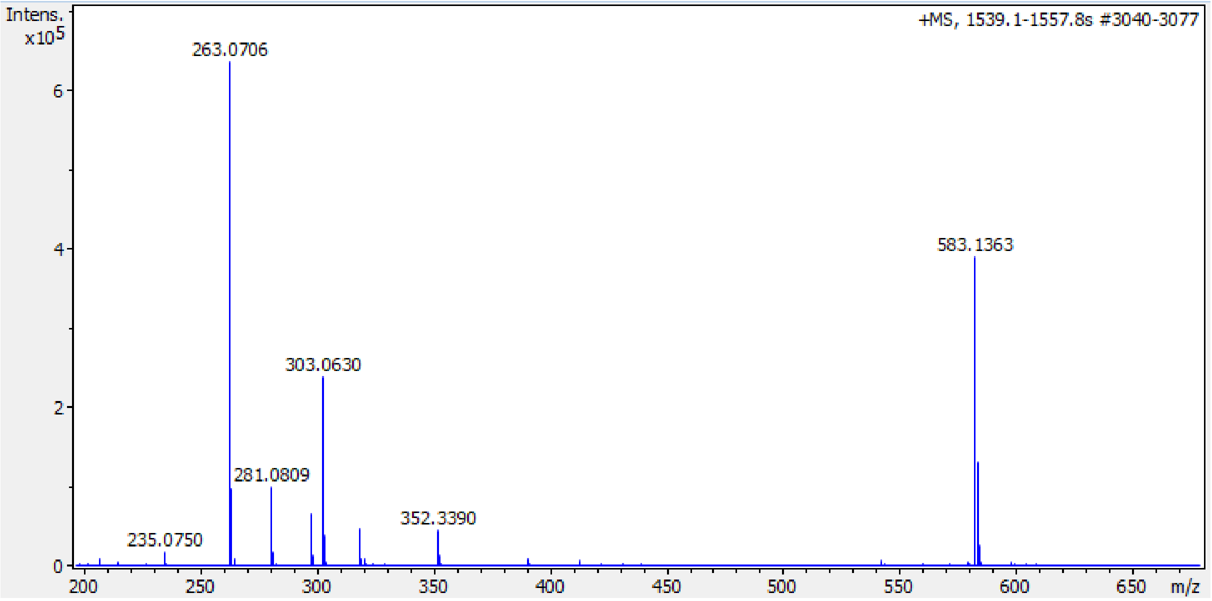

^{1}H (top) and ^{13}C (bottom) NMR spectra for compound **3** (THF-d_8, 30°C)

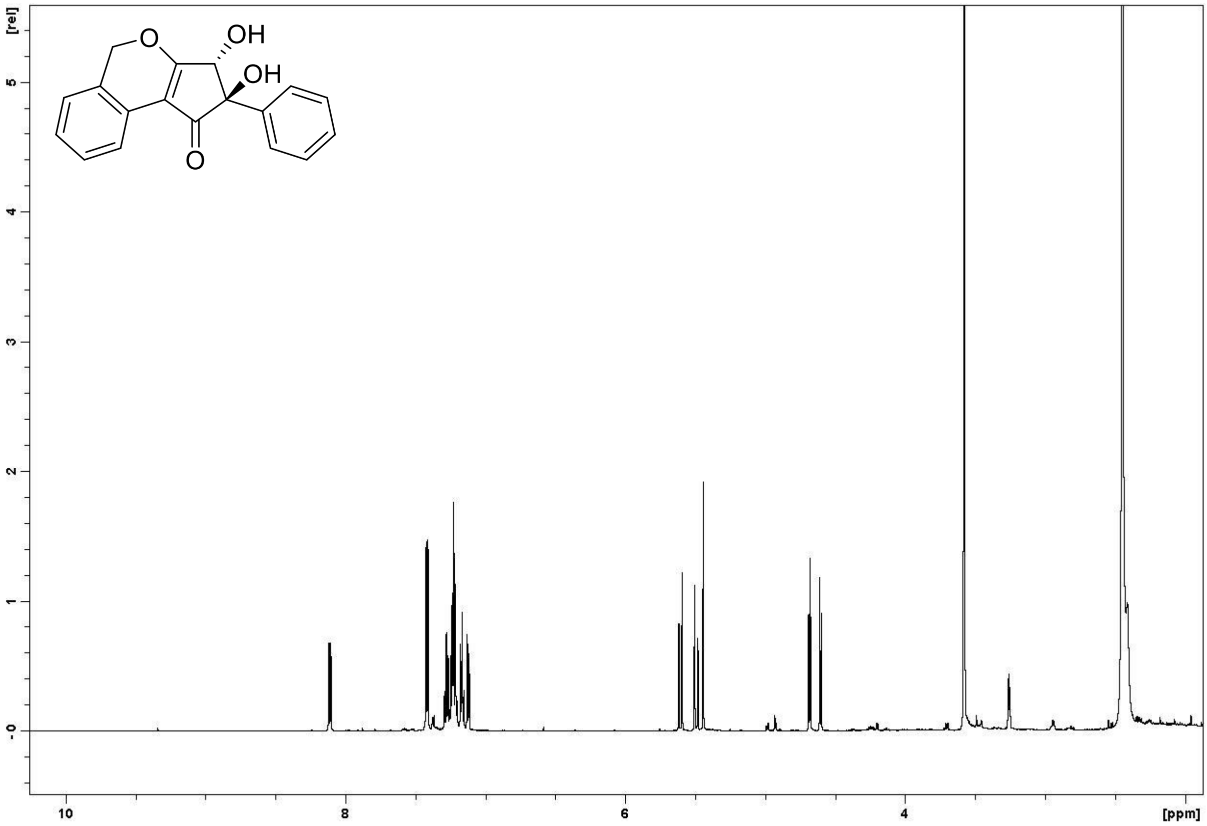

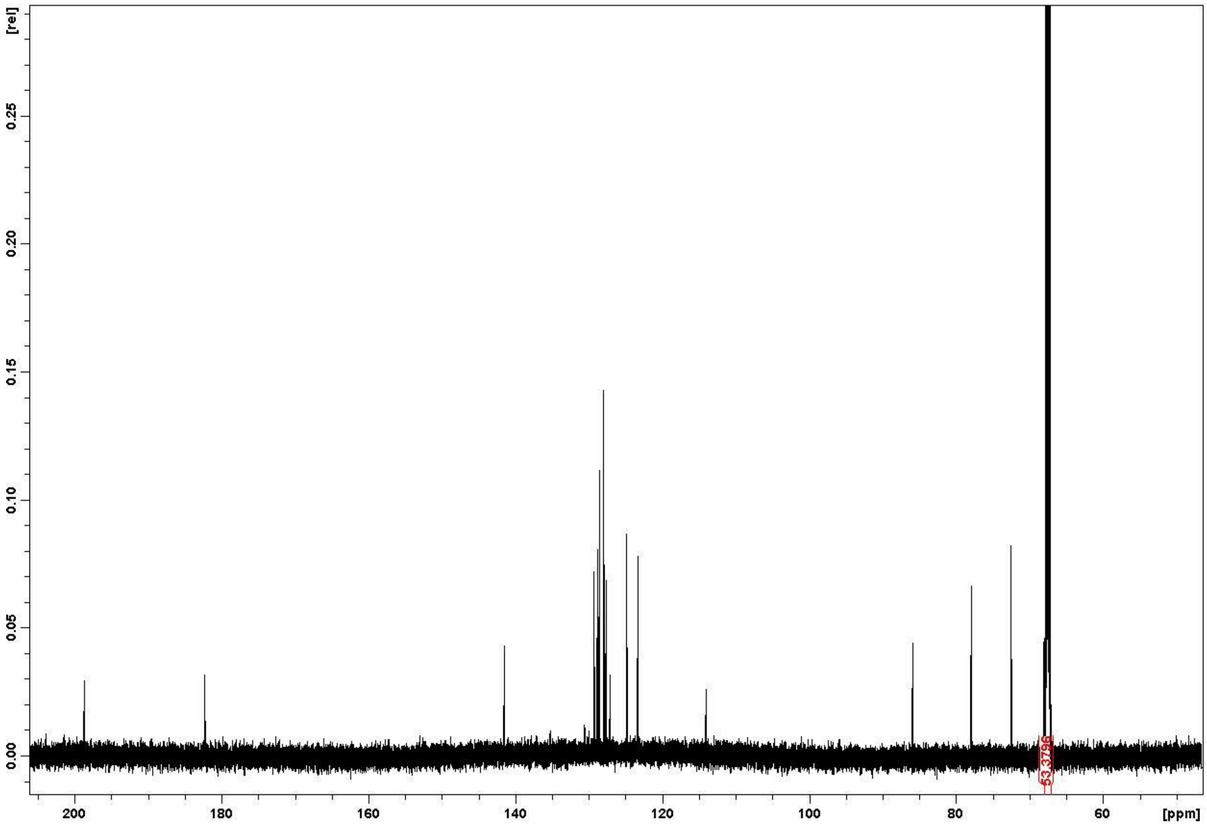

COSY (top) and HSQC (bottom) NMR spectra for compound **3** (THF-d_8, 30°C)

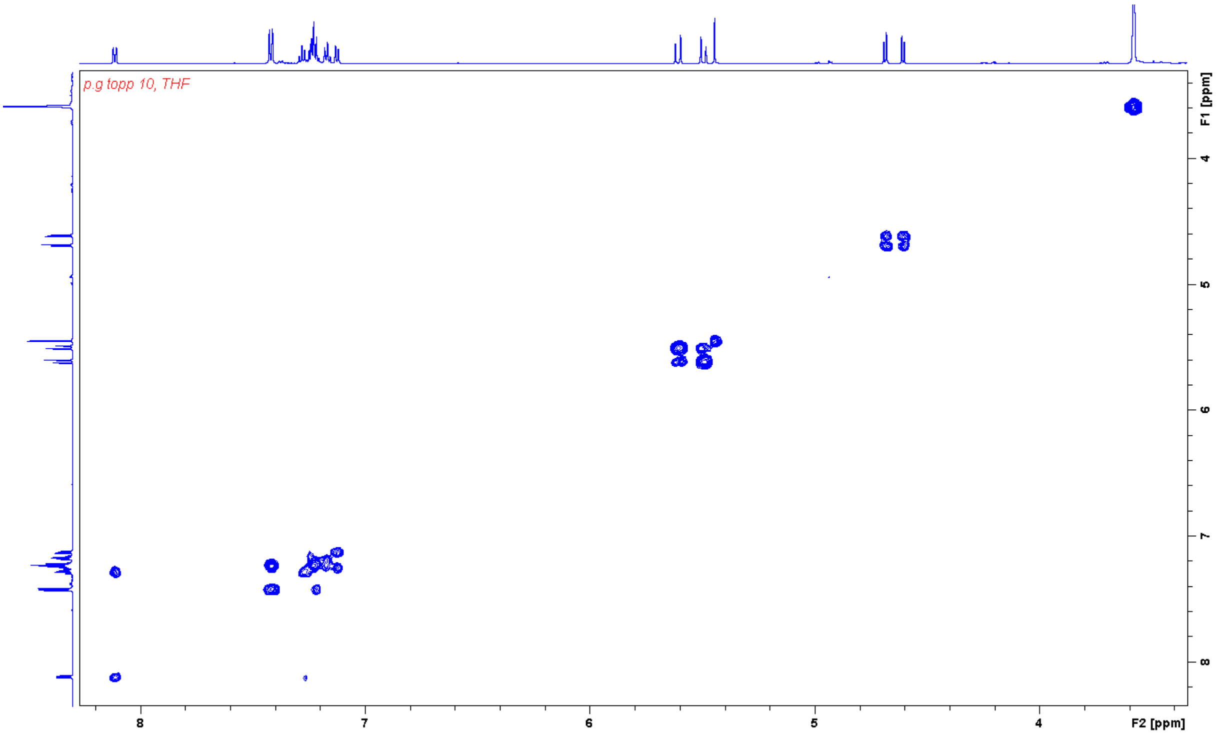

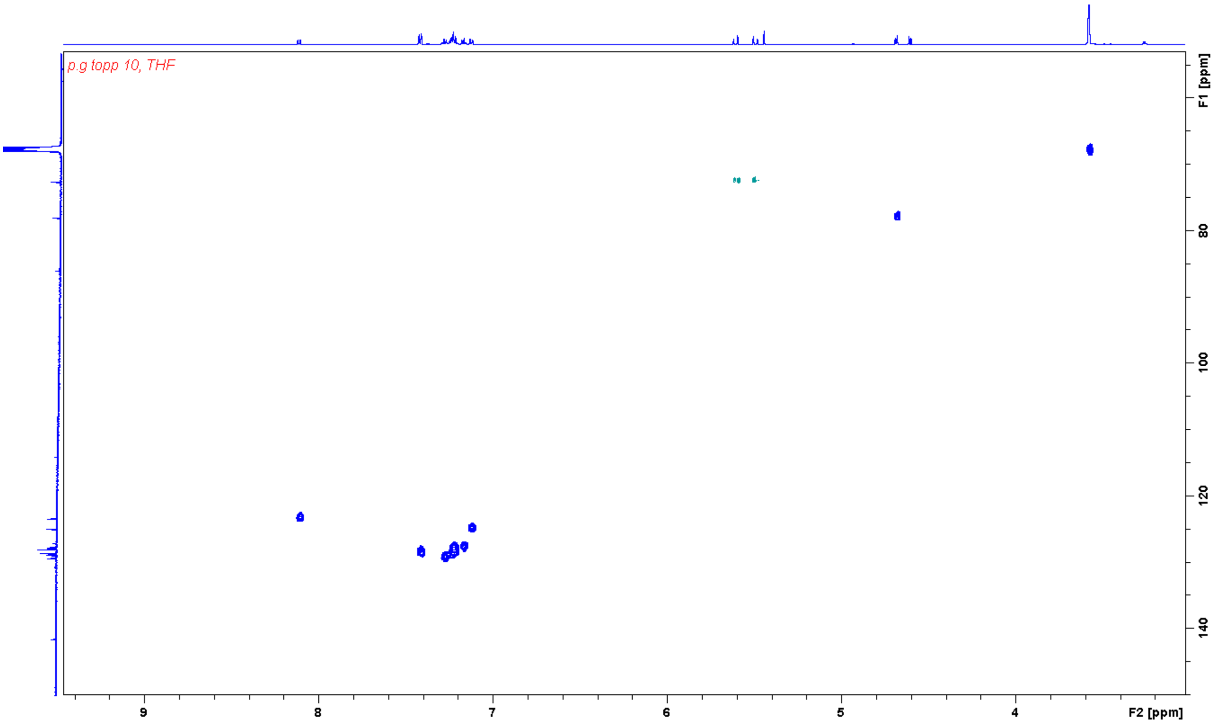

HMBC (top) and ROESY (bottom) NMR spectra for compound **3** (THF-d_8, 30°C)

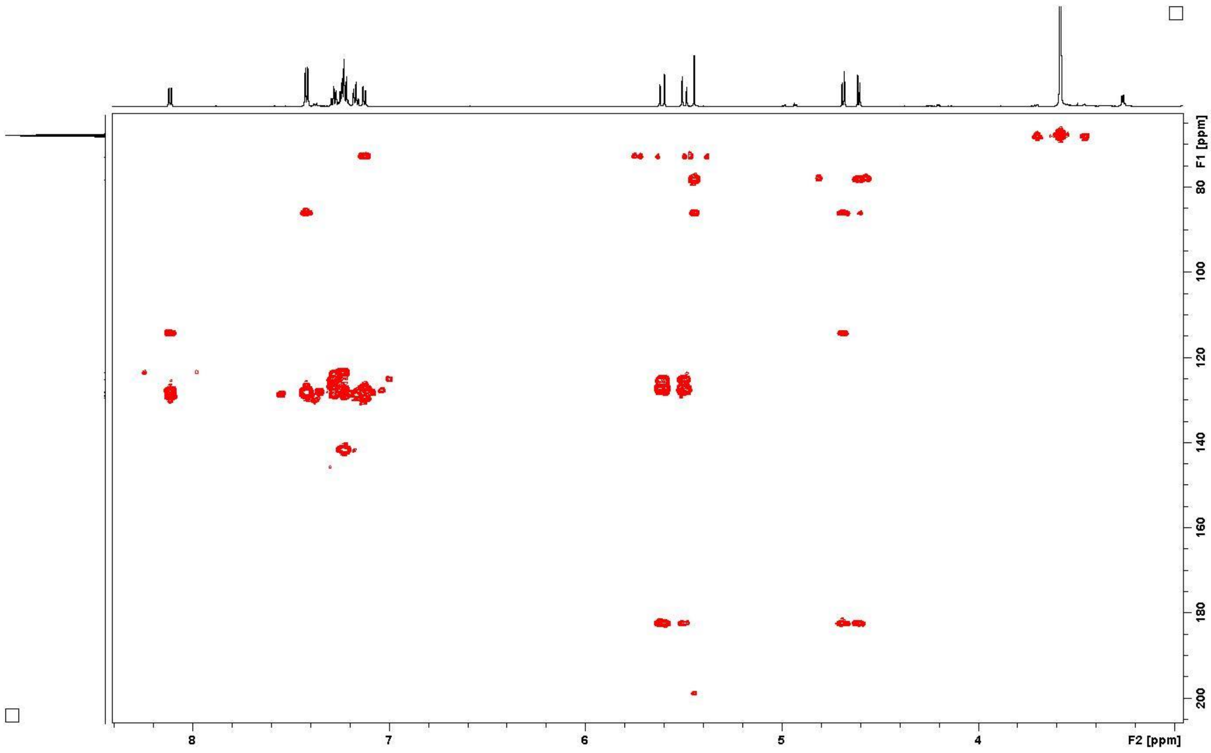

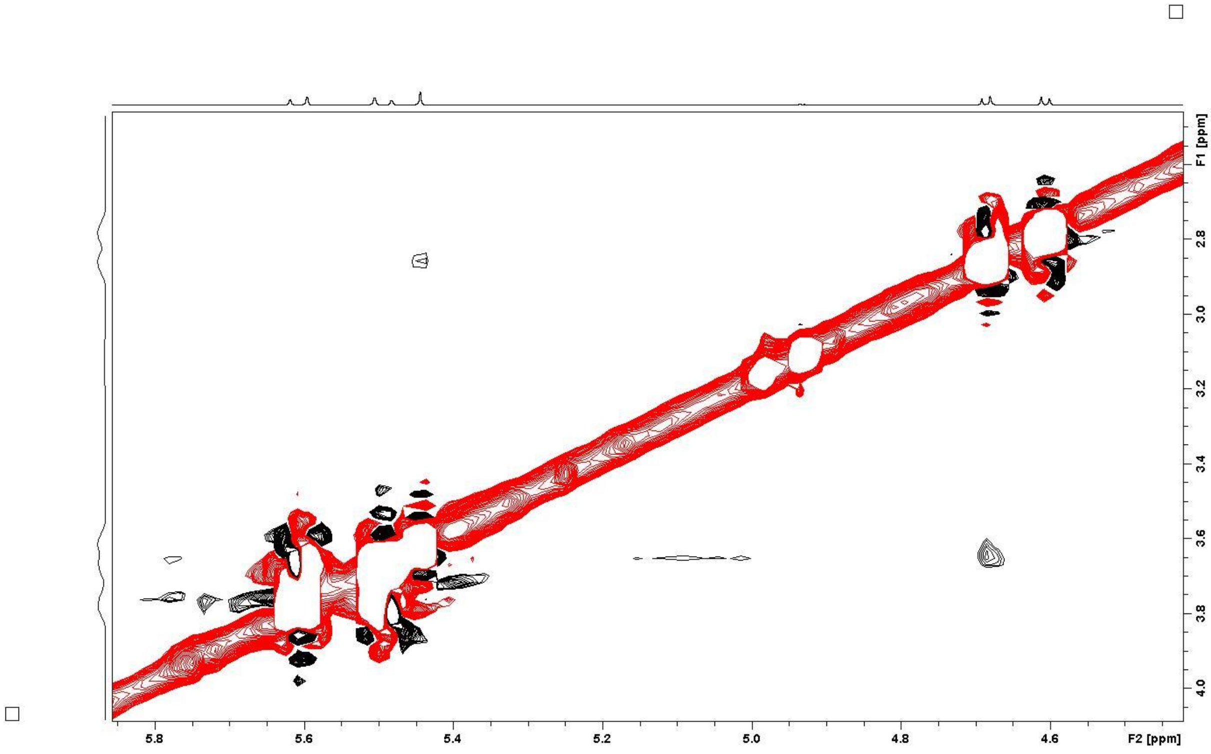

Mass spectrum for compound **3**. Major ions (from left to right): $[M+H-H_2O]^+$, $[M+H]^+$, $[M+Na]^+$and $[2M+Na]^+$.

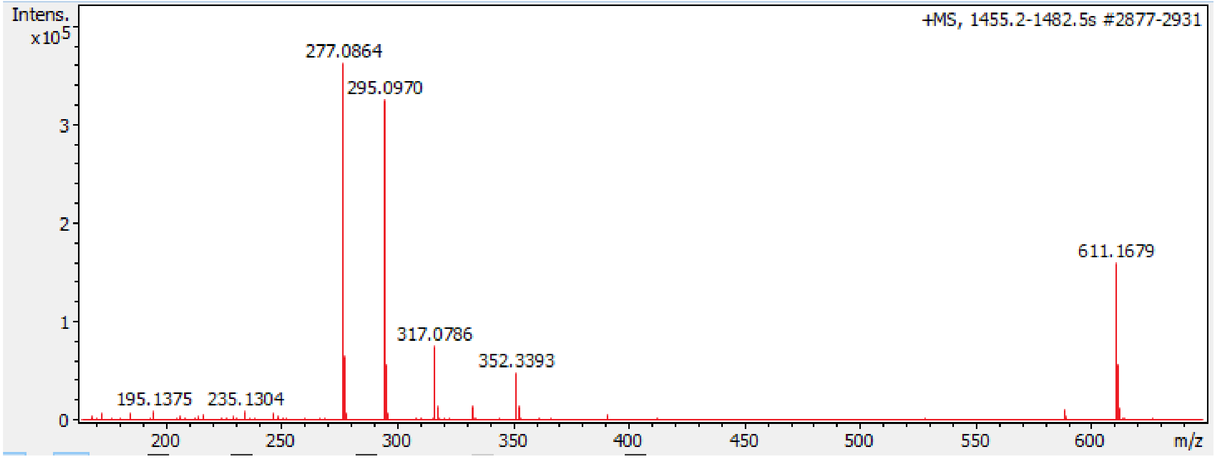

^{1}H (top) and ^{13}C (bottom) NMR spectra for compound **4** (acetone-d_6, and methanol-d_4, respectively, 30°C)

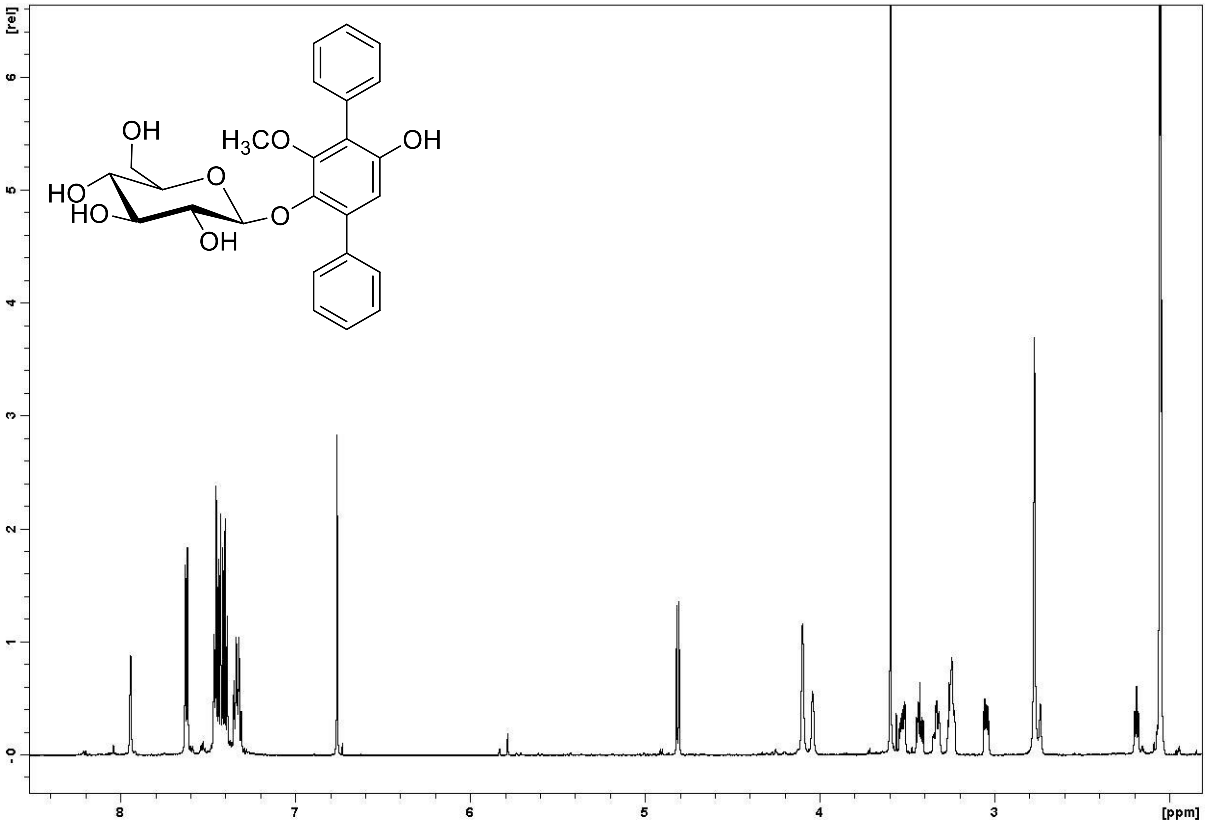

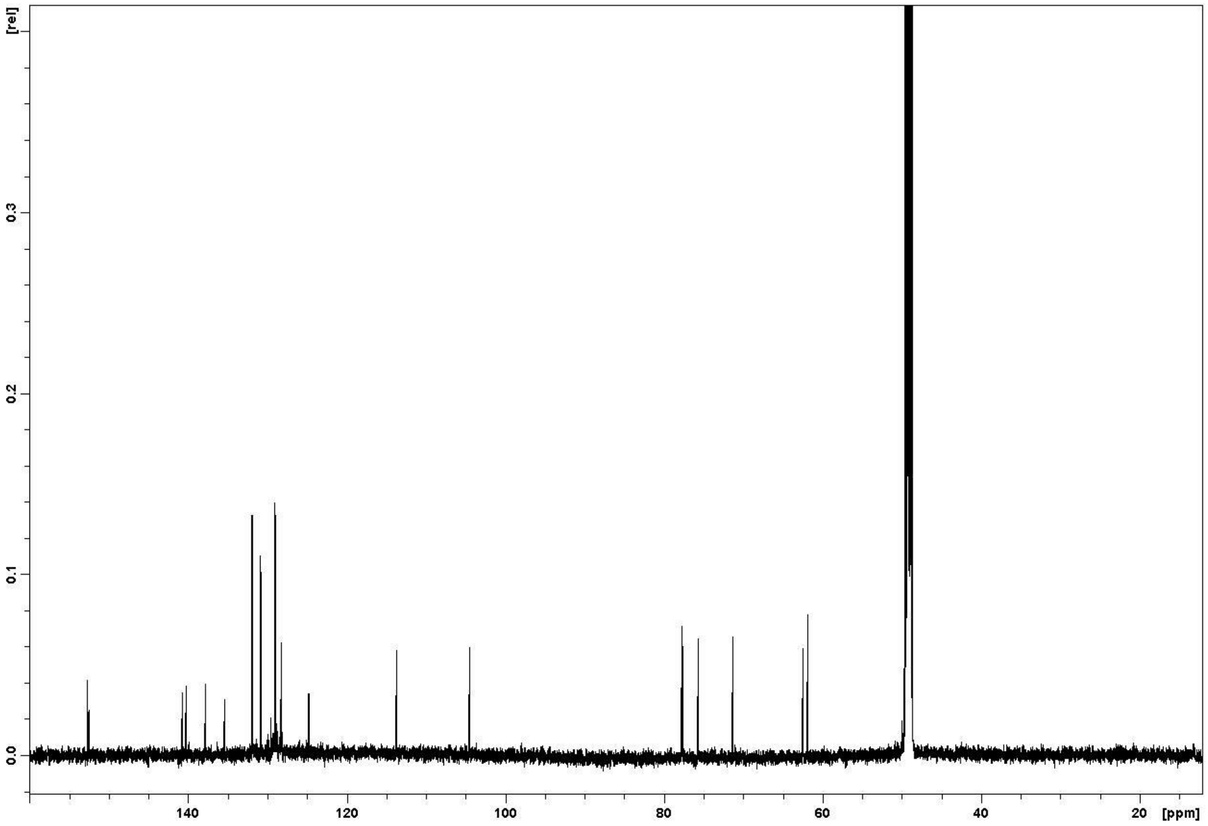

COSY (top) and HSQC (bottom) NMR spectra for compound **4** (acetone-d_6, 30°C)

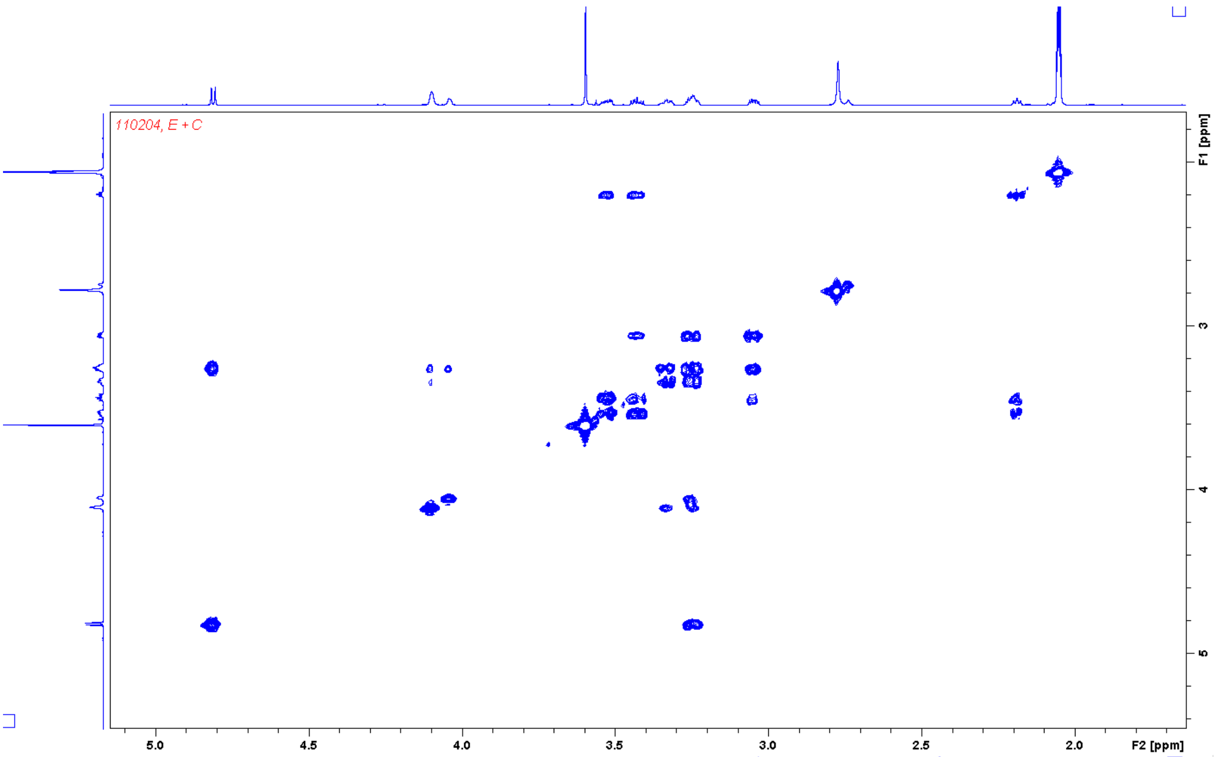

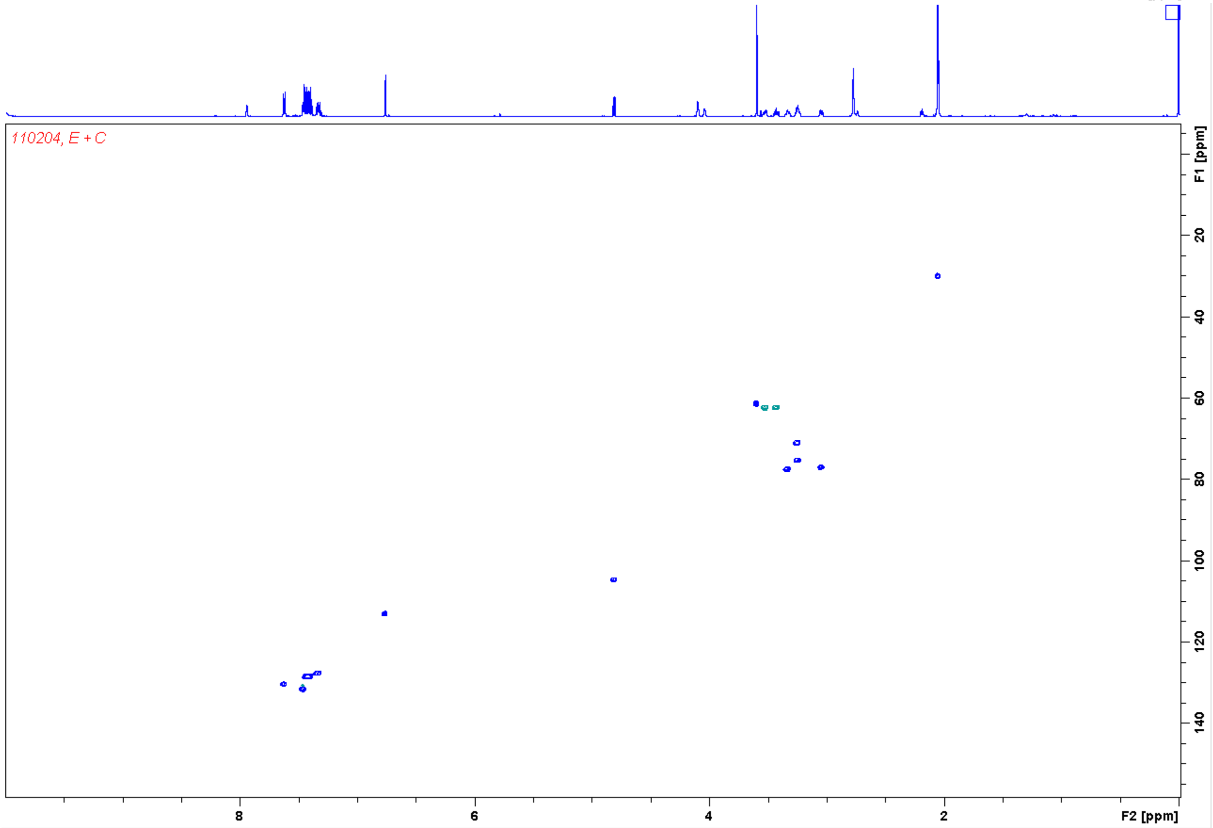

HMBC (top) and ROESY (bottom) NMR spectra for compound **4** (acetone-d_6, 30°C)

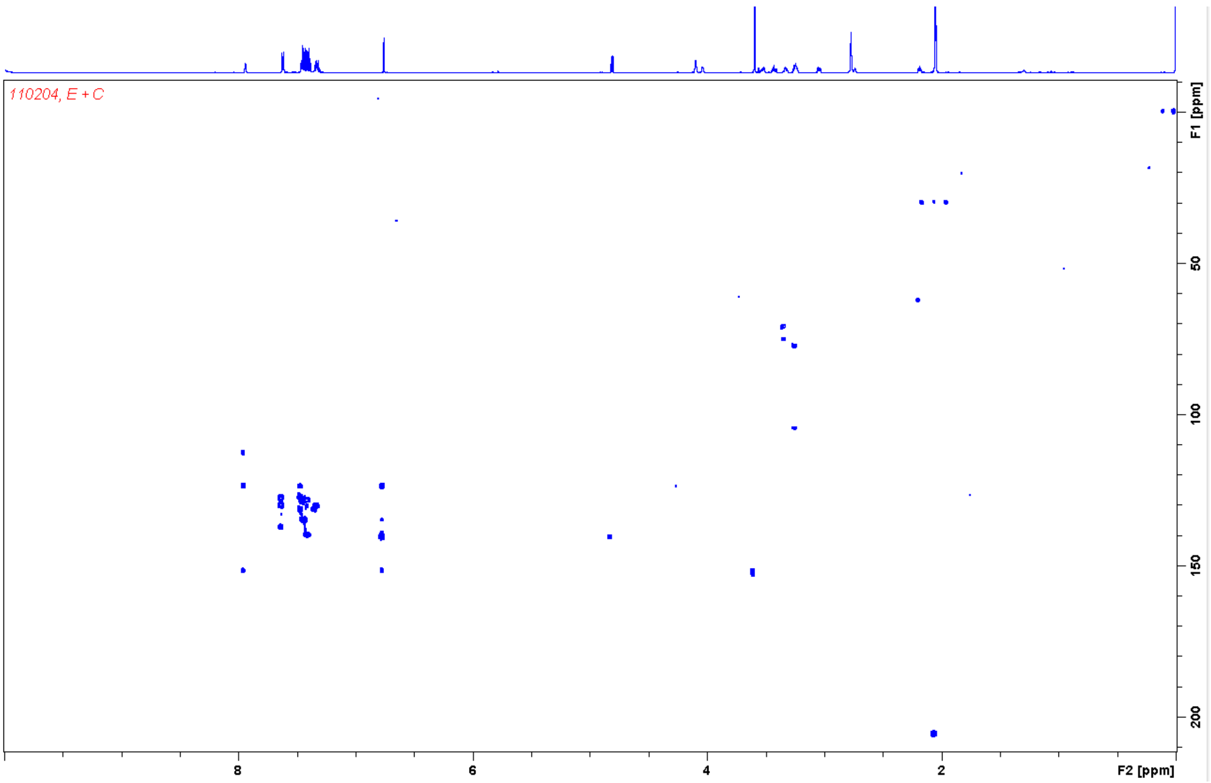

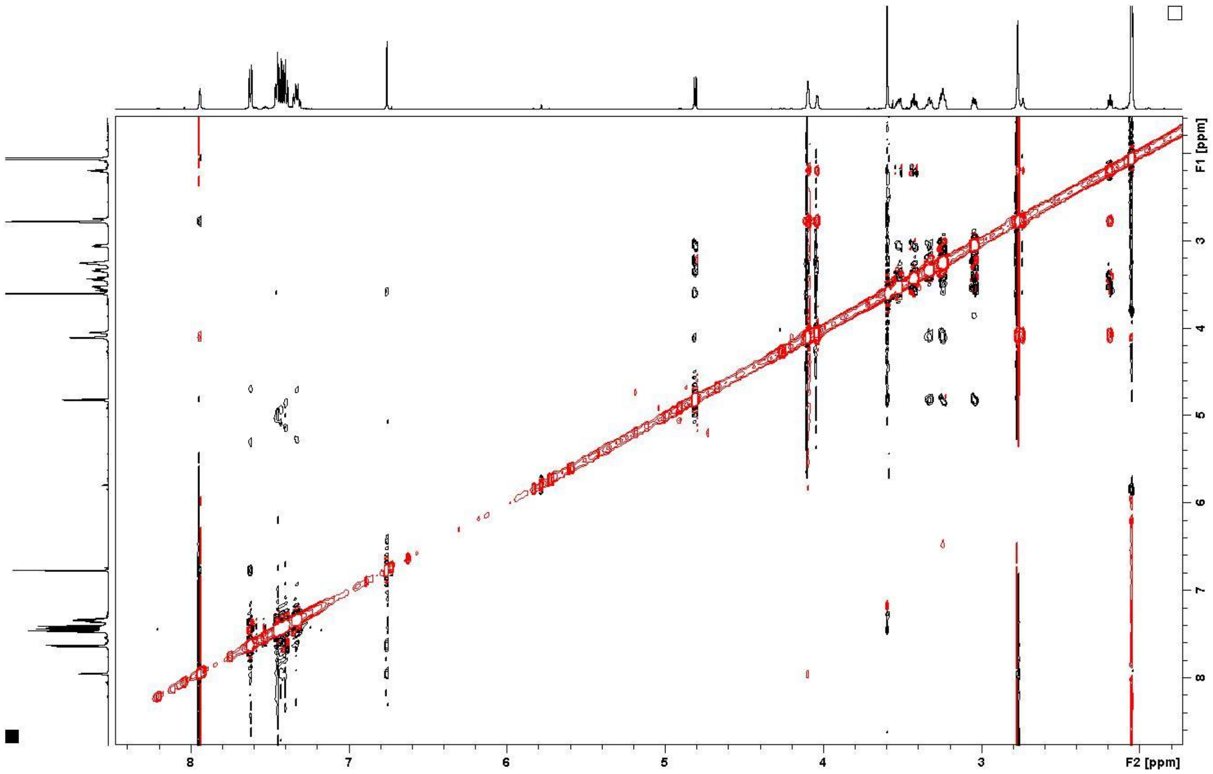

Mass spectrum for compound **4**. Major ions (from left to right): [M+H-Glc]$^+$, [M+NH_4]$^+$, and [M+Na]$^+$.

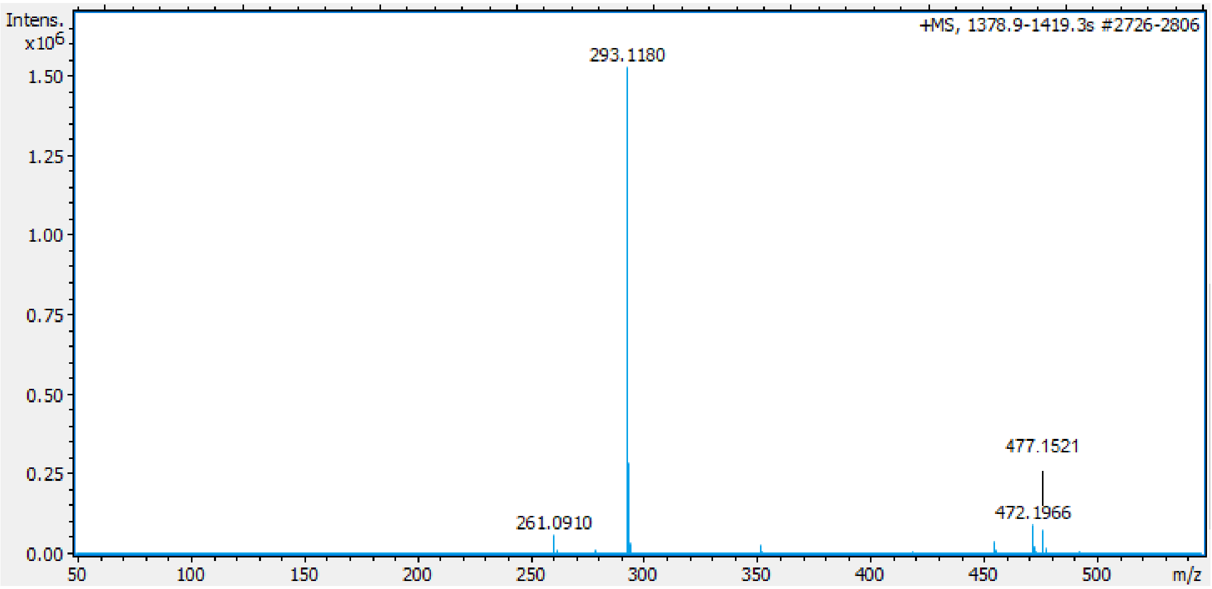

Supplement: Supplementary file 1 [file molecules-23-01417-s001.pdf]
